# Recent Advances in Non-Noble Metal Electrocatalysts for Hydrogen Evolution Reaction in Water Splitting

**DOI:** 10.3390/nano15141106

**Published:** 2025-07-16

**Authors:** Aiyi Dong, Zifeng Li, Yinhua Ma, Weimin Liao, Fengjiao Zhao, Xun Zhang, Honglin Gao

**Affiliations:** 1 School of Science, Dalian Maritime University, Dalian 116026, China; aiyidong@dlmu.edu.cn; 2 State Key Laboratory of Catalysis, Collaborative Innovation Center of Chemistry for Energy Materials, Dalian Institute of Chemical Physics, Chinese Academy of Sciences, Dalian 116023, China; zifengli@mail.ustc.edu.cn; 3 Marine Engineering College, Dalian Maritime University, Dalian 116026, China; lwm20021202@dlmu.edu.cn; 4 Transportation Engineering College, Dalian Maritime University, Dalian 116026, China; zx2220223740@dlmu.edu.cn

**Keywords:** hydrogen evolution reaction, non-noble metal electrocatalysts, synthesis methods, catalytic performance, reaction mechanism

## Abstract

Electrochemical water splitting is an efficient and eco-friendly method for hydrogen production, offering a sustainable energy solution. Currently, the noble metal platinum is considered to be the most efficient catalyst for electrochemical hydrogen evolution reactions (HERs). Due to the scarcity and high cost of noble metal materials, there is an urgent need to find abundant and cost-effective non-noble metal catalysts to reduce the overpotential of HERs. In recent years, significant scientific advancements have been reported in non-noble metal HER catalysts. This review categorizes and reviews the recent non-noble metal HER catalysts and their reaction mechanisms. An exhaustive overview of proven effective catalyst categories is provided, offering early-career researchers a panoramic understanding of this dynamic research field. Finally, we address current challenges and future directions in this field to encourage further research efforts and the development of non-noble metal catalysts.

## 1. Introduction

The excessive exploitation and utilization of non-renewable fossil fuels have caused serious resource depletion, environmental pollution, and ecological imbalance. In the face of pressure on resources and the environment, it is imperative to develop alternative sources of energy that are clean, efficient, and sustainable. Hydrogen (H_2_), with the properties of cleanliness, recyclability, low density, high calorific value, and storability, is widely used in various industries including aerospace, electronics, and transportation, and is considered to be a high-quality alternative energy to traditional fuels for the future [1,2,3,4]. Currently, high-temperature hydrogen production from fossil fuels is still the main approach to industrial hydrogen production [5]. This is contrary to energy substitution strategies in the context of sustainable development. The water electrolysis strategy for hydrogen production at room temperature uses the earth’s abundant water resources as the raw material, and the reaction products are only hydrogen and oxygen. With the electricity provided by solar, wind, and nuclear energy to drive this reaction, it can achieve zero carbon dioxide emission throughout the whole process [6,7]; thus, water electrolysis is regarded as a clean and highly efficient technology for hydrogen production.

Water electrolysis includes two half-reactions: the cathodic hydrogen evolution reaction (HER) and the anodic oxygen evolution reaction (OER). In theory, the thermodynamic potential for water electrolysis is 1.23 V (vs. the reversible hydrogen electrode, RHE). However, due to the various influences such as polarization and internal resistance, the electrode potential of the actual reaction is much higher than 1.23 V. Therefore, efficient electrocatalysts are required to accelerate the reaction kinetics and reduce the overpotential. Pt-group noble metals are the most efficient catalysts for hydrogen evolution [8,9]. However, the high scarcity and price of noble metals have severely hindered their application in large-scale industrialized hydrogen production [10], leading researchers to turn their focus to developing non-noble metal catalysts that are abundant and inexpensive. In recent years, with the continuous optimization of synthesis methods and the wide application of advanced characterization techniques, nanoscience and technology have developed by leaps and bounds, and a variety of innovative non-noble metal catalysts have been developed, leading to a blossoming research landscape.

This paper presents a comprehensive and in-depth review of the state-of-the-art non-noble metal catalysts for HERs. We systematically classify and summarize the latest high-performance catalysts reported in recent years, covering a diverse range of materials including transition metal compounds, metal–organic frameworks (MOFs) and their derivatives, alloys and intermetallic compounds, high-entropy alloys and high-entropy oxides, as well as heterostructure catalysts. For each category, we conduct a detailed analysis of synthesis strategies, advanced characterization techniques, performance evaluation methods, and optimization approaches. Based on the results, the challenges are analyzed and future research directions are sought, with the aim of realizing the best practical applications.

## 2. Fundamentals of Hydrogen Evolution Reaction Through Water Electrolysis

In the standard case of 1.0 atm at 25 °C, the overall reaction of water electrolysis can be expressed as follows [11]:

(1) $$2H_{2}O\to O_{2}+2H_{2}$$

At this point, the theoretical thermodynamic equilibrium potential for the reaction is 1.23 V, regardless of the acidity or alkalinity of the electrolytes. The HER reaction is a two-electron-transfer process that occurs at the cathode surface through two different mechanisms and three possible reactions [12]. In acidic electrolytes (e.g., sulfuric acid or hydrochloric acid), the corresponding HER process can be expressed as follows [13,14]:

1. Volmer reaction: The protons of H_3_O^+^ in the electrolyte are captured by the electrons on the catalyst surface, forming adsorbed hydrogen (m − H) on the catalyst surface.

(2) $$H_{3}O^{+}+e^{-}\to m-H+H_{2}O$$

2. Heyrovsky reaction: This adsorbed hydrogen atom couples to a proton and an electron to form a hydrogen molecule.

(3) $$H_{3}O^{+}+e^{-}+m-H\to H_{2}+m+H_{2}O$$

3. Tafel reaction: Adjacent adsorbed hydrogen atoms couple to form a hydrogen molecule.

(4) $$2m-H\to H_{2}$$

where m represents the active site of the catalyst. Whereas in alkaline or neutral electrolytes, the hydrogen evolution reaction can be expressed as follows [10,15,16]:

1. Volmer reaction: Water molecule provides protons that combine with electrons to form adsorbed hydrogen, replacing the role of H_3_O^+^ in acidic solutions.

(5) $$H_{2}O+e^{-}+m\to m-H+OH^{-}$$

2. Heyrovsky reaction: The adsorbed hydrogen atom attracts both a water molecule and an electron to produce a hydrogen molecule.

(6) $$m-H+H_{2}O+e^{-}\to m+H_{2}+OH^{-}$$

3. Tafel reaction: Like in acidic conditions, two adjacent adsorbed hydrogen atoms combine to form a hydrogen molecule.

(7) $$2m-H\to H_{2}$$

Under alkaline conditions, the process of a HER will be slower than in acidic media; this is due to the dissociation of water molecules before the formation of m−2H [17]. In alkaline electrolyte, it is the strong covalent bond in H-O-H that needs to be broken, rather than the weak covalent bond in H_3_O^+^ in acidic electrolyte [14,18]. Whether in acidic, alkaline, or neutral electrolytes, the HER begins with the adsorption of protons on the catalyst surface, which is called the Volmer reaction. Subsequently, hydrogen molecules may be generated by two possible routes, one is an electrochemical desorption process, the Heyrovsky reaction, and the other is a chemical desorption process, the Tafel reaction [19]. Irrespective of the routes of hydrogen evolution reaction, the adsorption and desorption of m-H are carried out throughout. Therefore, the free energy of hydrogen adsorption $\Delta G_{H^{*}}$ can be viewed as a marker for evaluating the hydrogen evolution performance of the catalyst [20]. The optimal catalyst $\Delta G_{H^{*}}$ for the hydrogen evolution reaction should be close to 0. Weak adsorption leads to poor proton interaction with the catalyst surface, while strong adsorption can severely hinder the desorption of hydrogen molecules [19].

The water electrolysis process under acidic conditions usually uses a proton exchange membrane (PEM) water electrolyzer, which has the advantages of rapid reaction kinetics and high energy utilization. However, the presence of acidic electrolyte limits the substitution of non-noble metal catalysts for noble metal catalysts due to the higher stability of noble metal catalysts, resulting in high hydrogen production costs. Electrolytic processes in alkaline electrolyte enable the large-scale application of non-noble metal catalysts compared to acidic electrolyte [18]. Improving the kinetics of HERs in alkaline electrolyte by using efficient non-noble metal catalysts is the direction that needs to be focused on at this stage of research [21].

## 3. HER Electrocatalysts Performance Evaluation Parameters

An ideal catalyst for HERs should have the following characteristics: (1) excellent charge transfer ability; (2) high intrinsic activity; (3) abundant active sites; (4) large electrochemical active surface area; (5) high reaction rate; (6) sufficient catalytic durability; and (7) adequately low material synthesis cost. In order to measure the advantages and disadvantages of different catalysts, various electrochemical performance evaluation parameters for HERs have emerged, which have become a benchmark in the continuous development of the HER.

### 3.1. Overpotential

During water electrolysis, the thermodynamic equilibrium potentials for the HER and OER are 0 V (vs RHE) and 1.23 V (vs RHE), respectively (both versus the reversible hydrogen electrode). The overpotential (*η*) is the difference between the actual voltage (*E*_i_) and the theoretical voltage (*E*_t_) required to maintain a certain current density (*j*) during the actual occurrence of the catalytic reaction. The formula for the calculation of overpotential is as follows:

(8) $$\eta =E_{i}-E_{t}$$

The overpotential during water electrolysis consists of three components: activation overpotential, concentration overpotential, and resistance overpotential. The activation overpotential is determined by the intrinsic properties of the electrocatalyst, and the improvement of the catalyst is oriented toward the reduction of the activation overpotential. Concentration overpotential is attributed to a decrease in the concentration of the electrolyte near the electrode surface when the electrode reaction occurs, and such overpotential can be eliminated to some extent by stirring the electrolyte. For the three-electrode system, resistive overpotential is generated by the internal resistance of the solution between the working and reference electrodes, the wire resistance, and the contact point resistance [22]. The voltage caused by internal resistance can be subtracted from the overpotential data by IR compensation [19]. The value of the overpotential is obtained through Linear Sweep Voltammetry (LSV), where a smaller overpotential at the same current density or a higher current density at the same potential indicates better performance of the catalyst. In practical studies, to facilitate the comparison of different types of catalysts for HERs, the overpotential value at some specific current density (e.g., 10 mA cm^−2^ or 100 mA cm^−2^) is used as a criterion.

### 3.2. Tafel Slope

The Tafel slope can be found from the Tafel equation:

(9) $$\eta =a+b\log\left|j\right|$$

where *η*, *b*, and *j* represent the HER overpotential, Tafel slope, and current density, respectively. As an important parameter for evaluating the reaction kinetics of the catalyst, the numerical magnitude of the Tafel slope can determine the rate-limiting step of the HER. Specifically, the hydrogenolysis reaction in the alkaline electrolyte solution contains the Volmer, Heyrovsky, and Tafel steps, which correspond to theoretical Tafel slopes of about 120 mV dec^−1^, 40 mV dec^−1^, and 30 mV dec^−1^ [23]. When the value of *η* is 0, the exchange current density *j*_0_ can be found by substituting it into the Tafer equation, where a larger exchange current density and a smaller Tafer slope represent higher catalytic activity of the catalyst [24].

### 3.3. Faradaic Efficiency

Faradaic efficiency is defined as the efficiency of electron transfer provided by an external circuit, and it can also be denoted as the ratio of actual hydrogen production to theoretical hydrogen production. The actual hydrogen production can be measured by gas chromatography, and the theoretical hydrogen production can be calculated by integrating constant current or constant potential electrolysis [25]. The closer the Faradaic efficiency is to 1, the less energy is lost during the reaction or the fewer by-products are produced.

### 3.4. Electrochemical Active Surface Area (ECSA)

The electrochemical active surface area is the effective area participating in the electrochemical reaction, which can reflect the exposure of active sites on the surface of the catalyst. The electrochemically active surface area is proportional to the double layer capacitance (*C*_dl_) of the electrocatalyst. The electrochemically active surface area is generally calculated by plotting cyclic voltammetry curves obtained in the non-Faradaic reaction zone.

### 3.5. Stability

Stability is also an important index for the evaluation of the catalyst’s superiority, reflecting the ability of the tested catalytic material to maintain catalytic activity when undergoing prolonged catalytic reactions. The chronoamperometry method, chronopotentiometry method, and cyclic voltammetry method are all commonly used for stability testing [19]. While the first two methods are performed by observing how stable the voltage or current is over a long time range, cyclic voltammetry requires observing how much the polarization curves overlap before and after several CV scans. In general, stable catalysts need to be able to withstand a current density of not less than 10 mA cm^−2^ for more than 10 h, or more than 5000 rounds of CV cycling, without changing their catalytic activity [12]. To ensure the application of catalysts in industrial hydrogen production, long-term stability tests under high OH^−^ concentration, high temperature, and high current density are indispensable. Currently, the active sites of most non-noble metal catalysts decay faster than those of noble metals, limiting their long-term application in complex industrial environments [26]. Although the cost of non-noble metal catalysts is approximately 90% lower than that of platinum-group noble metals, frequent replacement will increase comprehensive costs [27]. Therefore, the development of highly stable non-noble metal catalysts is imperative.

### 3.6. Catalyst Loading and Electrolyte pH

The catalyst loading and the pH of the electrolyte are key factors affecting the HER process in water electrolysis. These two factors significantly influence HER performance through mechanisms such as regulating reaction kinetics, affecting the degree of exposure of active sites, and influencing the adsorption behavior of reactants.

Within a certain range, there is a positive correlation between catalyst loading and performance [28]. At low loadings (typically less than 1 mg cm^−2^), catalyst particles exhibit good dispersibility, allowing abundant exposure of active sites such as edges or defects, which results in high catalytic efficiency per unit mass. However, excessively low loading may lead to insufficient contact area between the catalyst and the substrate, reducing electron conduction efficiency and compromising mechanical stability. Cost issues related to high catalyst loading are often not factored into the design of non-noble metal catalysts. However, excessively high loading (typically >5 mg cm^−2^) tends to cause catalyst agglomeration or pore blockage, which not only shields active sites but also hinders electrolyte diffusion.

The pH value of the electrolyte influences multiple aspects of the HER, including the reaction mechanism, catalyst activity, and mass transfer process [29]. First, as mentioned in Chapter 2, electrolytes with different acid–base properties correspond to distinct proton sources and rate-determining steps. The HER under alkaline conditions requires overcoming the high energy barrier of water dissociation. Second, OH^−^ in alkaline media may competitively adsorb active sites or induce catalyst reconstruction, inhibiting HER activity. In solutions without buffering or with insufficient buffering capacity, the consumption of H^+^ causes a local pH increase, leading to a slowdown in HER kinetics. Finally, the interfacial electric field in alkaline conditions is stronger, with obvious orientational polarization of water molecules, increasing the reorganization energy and hindering charge/proton transfer [30].

## 4. Non-Noble Metal HER Electrocatalysts

### 4.1. Transition Metal Compound-Based Catalysts

#### 4.1.1. Transition Metal Carbides/Nitrides

Transition metal atoms differ from noble metals in their electronic structure and tend to exhibit a stronger hydrogen adsorption strength than noble metals, leading to hydrogen desorption becoming the rate-limiting step in the HER reaction [31]. When small-radius nonmetallic atoms form compounds with various transition metal elements, their interactions lead to the contraction of the d-band of the transition metal, which moves the center of the d-band upward to the Fermi energy level, and this phenomenon is beneficial for obtaining fast desorption performance in HERs, making the performance similar to that of the noble metal Pt [31]. Typical examples of such transition metal–nonmetal combinations include transition metal carbides, nitrides, phosphides, chalcogenides, borides, and oxides. Among them, transition metal carbides and transition metal nitrides perfectly combine the characteristics of ionic crystals, covalent compounds, and transition metals, resulting in simple crystal structures, outstanding hardness, as well as remarkable electrical conductivity and stability [32,33].

Annealing under an NH_3_ atmosphere is a common form of obtaining nitrides. For example, sequential annealing of hydrothermally generated NiMoO nano-array precursors in NH_3_ and Ar/H_2_ atmospheres yielded surface-roughened NiMoNH [34]. Such nitride nanopillar arrays in alkaline electrolyte had a lower overpotential (183 mV for 100 mA cm^−2^) and Tafel slope (146 mV dec^−1^) than NiMOH catalysts (224 mV for 100 mA cm^−2^ and 161 mV dec^−1^) that were annealed only in an Ar/H_2_ atmosphere.

**Figure 1:**
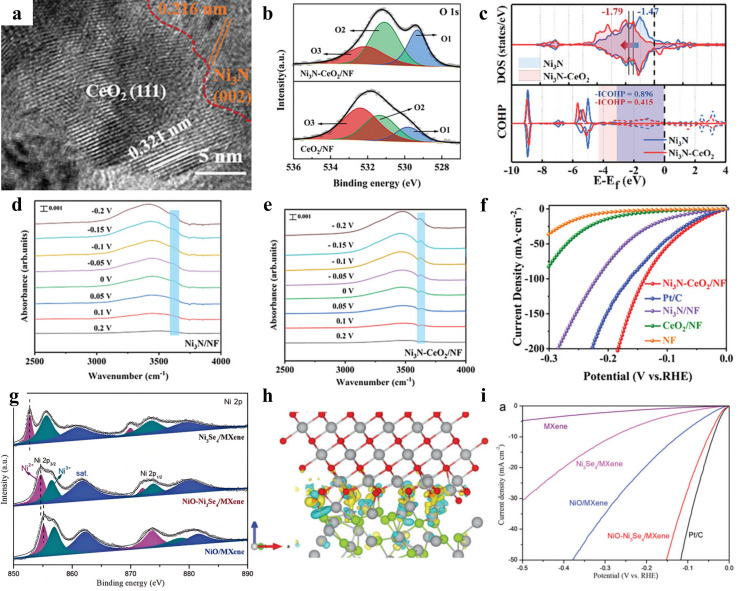
(**a**) TEM of Ni_3_N-CeO_2_/NF at 5 nm. (**b**) O1 s XPS spectra of Ni_3_N-CeO_2_/NF and Ni_3_N/NF. (**c**) The DOS and COHP diagrams of the Ni site in Ni_3_N and Ni_3_N-CeO_2_. In situ attenuated total reflection surface-enhanced infrared absorption spectroscopy (ATR-SEIRAS) of (**d**) Ni_3_N/NF and (**e**) Ni_3_N-CeO_2_/NF. (**f**) LSV polarization curves of Ni_3_N-CeO_2_/NF, Ni_3_N/NF, CeO_2_/NF, NF, and Pt/C (20%). Reproduced with permission [36]. Copyright 2023, Wiley-VCH. (**g**) High-resolution XPS spectra of Ni 2p. (**h**) The charge transfer difference for NiO-Ni_3_Se_4_ heterostructure shows the regions of charge accumulation (yellow) and charge depletion (cyan). (**i**) LSV curves of NiO-Ni_3_Se_4_/MXene, NiO/MXene, Ni_3_Se_4_/MXene, bare MXene, and Pt/C. Reproduced with permission [42]. Copyright 2023, Wiley-VCH.

To solve the problem of the poor adsorption/desorption performance of single nitrides, numerous heterostructures have been explored, among which the use of electron-attracting methods to modulate the electronic structure of nitrides and to change the d-band center energy level were most common. Wang and coworkers synthesized a Mo_2_N/Ni_0.2_Mo_0.8_N dual-phase nitride nanoribbon heterostructure by hydrothermal methods and the nitridation process [35]. By rationally controlling the content of Ni, the structural collapse and metal-phase segregation in the nitridation process can be effectively solved; at the same time, the Ni^2+^ can also be controllably inserted into the matrix of the molybdenum nitride compound. Ding et al. constructed a Ni_3_N-CeO_2_/Nickel Foam(NF) heterostructure (Figure 1a) with ample oxygen vacancies [36]. X-ray photoelectron spectroscopy (XPS) spectra demonstrated that the presence of Ni_3_N helped with obtaining more oxygen vacancies (Figure 1b). The introduction of CeO_2_ decreased the electron density and d-band central energy level of the Ni sites in Ni_3_N (Figure 1c). The presence of the heterogeneous structure promoted the charge redistribution between the two phases. It is observed from attenuated total reflection surface-enhanced infrared absorption spectroscopy (ATR-SEIRAS) that the peaks of free water in this heterogeneous structure are stronger in intensity and higher in frequency compared with those of pure Ni_3_N (Figure 1d,e). This suggested that the charge redistribution made the water molecules more accessible to the active site, which enhanced the adsorption of water on the catalyst and thus accelerated the Volmer step in the HER process. The charge redistribution led to a decrease in the electron density of the Ni site in Ni_3_N, and density functional theory (DFT) corroborated that its antibonding state was below the Fermi energy level, and this electron transfer weakened the adsorption strength of OH and H at the Ni site, which was conducive to the desorption of OH and H. The ease of OH desorption is likewise evidenced by the fact that the crystal orbital Hamiltonian population (COHP) has more antibonding orbital components below the Fermi energy level. Ni_3_N-CeO_2_/NF samples with a Ce/Ni molar ratio of 2:1 in a 1 M KOH electrolyte exhibited the best HER performance, requiring only 39 mV and 96 mV overpotentials to yield current densities of 10 mA cm^−2^ and 50 mA cm^−2^, respectively (Figure 1f). Similarly, the introduction of oxides in the MoO_2_/Mo_2_N heterostructure optimized the hydrogen desorption performance of Mo sites in molybdenum nitride, achieving an overpotential of 335 mV at an ampere-level current density of 1 A cm^−2^, along with a Faradaic efficiency of 99.8% [37]. There were numerous other similar examples of constructing heterostructures with nitrides, effectively regulating the electronic structure at the heterogeneous interface [38,39,40,41].

Analogous to nitrides, transition metal carbides also suffer from high desorption energy barriers for adsorbed hydrogen; thus, electronic modulation of hydrogen adsorption sites in transition metal carbides has become a necessary step to improve their HER properties. Doping late transition metal elements in W_2_C can significantly reduce its charge transfer resistance and achieve better active site exposure; Ni-doped W_2_C (Ni-W_2_C) exhibited an overpotential of 88 mV at 10 mA cm^−2^ and a significantly higher value of hydrogen turnover frequency (TOF) than pristine W_2_C [43]. However, unlike the heterostructures that attract electrons in the aforementioned nitrides, the late transition metal elements have unpaired electrons and partially filled d-orbitals, which serve as electron donors after doping. Similar to the late transition metals, the doping of the non-transition metal Al can also serve to provide electrons for the carbides [44].

In recent years, an emerging class of two-dimensional carbides, nitrides, and carbonitrides (MXenes) have been synthesized and used for HERs. They stand out from traditional transition metal carbides/nitrides due to their high hydrophilicity, excellent electrical conductivity, and tunability of surface functionality [31,45,46]. The most precious feature of MXenes is their extraordinary hydrophilicity, which relies on the presence of abundant hydrophilic functional groups (e.g., -OH, -O, and -F) on the surface of MXenes. The presence of these functional groups ensures that MXenes are able to interact strongly with reactants, significantly reducing the aggregation and stacking of the reaction intermediates. The properties listed above make MXenes excellent substrates for the synthesis of HER catalysts. Yan et al. synthesized heterogeneous NiO-Ni_3_Se_4_ nanosheet arrays on two-dimensional Ti_3_C_2_T_x_ MXene, which exhibits good electrical conductivity [42]. The highly hydrophilic, high-conductivity MXene substrate and the heterogeneous structure formed by chemical coupling showed excellent synergistic effects. The MXene nanosheets facilitated Ni^2+^ homogeneous nucleation during synthesis and inhibited the occurrence of agglomerations in the subsequent oil bath process. Subsequent experiments and DFT calculations together corroborated that the asymmetric distribution of charge at the Ni_3_Se_4_ interface was caused by a strong interfacial built-in electric field resulting from robust electron coupling (Figure 1g,h). The negative-charge-enriched NiO optimized H^*^ adsorption, making the NiO-Ni_3_Se_4_/MXene catalyst exhibit a HER overpotential of 50 mV at 10 mA cm^−2^ (Figure 1i). Zhao and coworkers developed a surface modification strategy to assemble CoNi diatomic electrocatalysts on MXene substrates [47]. L-tryptophan acted as a highly effective surface modifier that could attach to the O terminal of Ti_3_C_2_T_x_ MXene through covalent Ti-O-C bonding, making the metal-O/metal-N bonds of Ti_3_C_2_T_x_ and L-tryptophan the basis for the stable attachment of Co and Ni atoms.

#### 4.1.2. Transition Metal Phosphides (TMPs)

**Figure 2:**
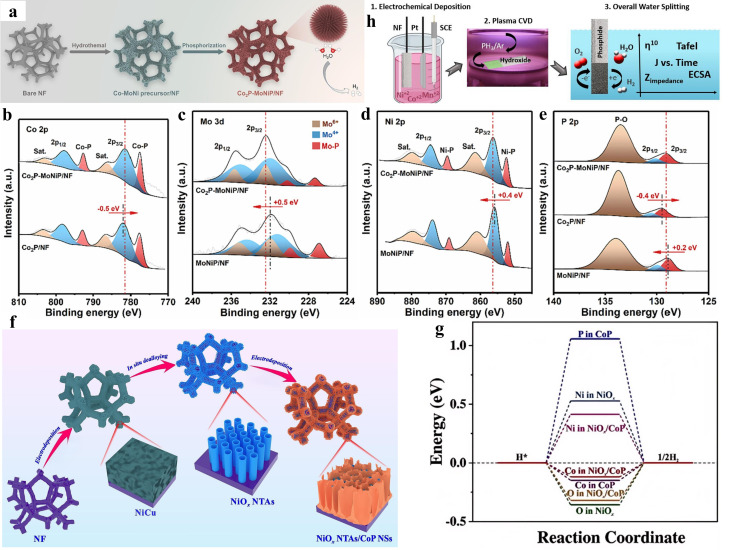
(**a**) Schematic diagram of Co_2_P-MoNiP/NF synthesis process. High-resolution XPS spectra of (**b**) Co 2p, (**c**) Mo 3d, (**d**) Ni 2p, and (**e**) P 2p for Co_2_P-MoNiP/NF, Co_2_P/NF, and MoNiP/NF [49]. Copyright 2023, Elsevier. (**f**) Schematic illustration of synthesis process of NiO_x_ NTAs/CoP NSs. (**g**) Free energy diagram for H^*^ adsorption. Reproduced with permission [21]. Copyright 2023, Elsevier. (**h**) Schematic of rational design of MnNiCo-P alloy on nickel foam as bifunctional catalyst for electrolytic water splitting. Reproduced with permission [54]. Copyright 2022, Zhengzhou University.

Due to the similarity in electronic structure to platinum, transition metal phosphides are one group of very promising HER catalysts. The phosphidation of catalytic materials often leads to better electrical conductivity and corrosion resistance. From another perspective, negatively charged phosphorus atoms possess an extremely strong ability to capture positively charged protons, which significantly increases HER activity. To verify the effect of phosphorus content on catalytic activity, Song et al. prepared CoNiP nanoarray-integrated electrodes with different phosphorus contents using ion etching and subsequent phosphidation procedures [48]. With the increase in phosphorus content, the value of the overpotential decreases and then increases, which indicates that a moderate amount of phosphorus can improve conductivity, but excessive phosphorus will cause unavoidable oxidation, and the thick oxidized layer will impede contact between the active site and the reactants, which in turn reduces catalytic efficiency. Zhao and coworkers synthesized sea-urchin-like Co_2_P-MoNiP/NF nanowires using a hydrothermal method and high-temperature phosphatization treatment (Figure 2a) [49]. These nanowires were made of a large number of aggregated nanoparticles, which ensured more contact area in the HER reaction. X-ray photoelectron spectroscopy (XPS) showed that the Co 2p and P 2p peaks of Co_2_P-MoNiP/NF were negatively shifted and the Mo 3d, Ni 2p, and P 2p peaks were positively shifted compared to Co_2_P/NF and MoNiP/NF, respectively (Figure 2b–e). This indicated that Co_2_P had a stronger ability to capture electrons and attracted some of the electrons from MoNiP, leading to electronic rearrangement at the interface of the heterostructure and enhanced hydrogen evolution activity. Simultaneously, the concentration ratio of Co and Mo was also taken into account, and the Co_2_P-MoNiP/NF with a Co^2+^/Mo^6+^ ratio of 3:1 exhibited the best homogeneity, the lowest overpotential (46 mV for 10 mA cm^−2^), and the smallest Tafel slope (49.3 mV dec^−1^). During the same period, a similar discovery was made by Zhang’s group [50]. They employed hydrothermal and phosphorization processes to synthesize the heterostructure Ni_2_P-CoCH/CFP on carbon fiber paper. The work functions (*Φ*), characterized by ultraviolet photoelectron spectroscopy (UPS), were calculated to be 5.87 eV and 6.38 eV for CoCH/CFP and Ni_2_P/CFP, respectively. The work function difference (Δ*Φ*) between them induced the creation of a built-in electric field (BEF), which resulted in Ni_2_P bringing the Gibbs free energy of hydrogen adsorption ( $\Delta G_{H^{*}}$) at the Ni sites closer to the ideal value of 0 eV by attracting electrons from CoCH. There have been numerous instances of preparing heterostructures of phosphides by thermal phosphatization methods similar to those described above, such as coating Fe_2_P nanoparticles on highly conductive Co_2_N surfaces [51], constructing orthorhombic crystalline CoSe_2_/amorphous CoP heterojunctions [52], preparing honeycomb-like heterogeneous and ultrathin Co_2_P-Fe_2_P nanosheets on the basis of CoFe Prussian blue analogue [53], and so on.

However, during the typical phosphidation process, toxic gases were often produced, which contradicted environmental protection strategies and threatened the safety of the experiments. Based on this fact, multiple strategies for the introduction of phosphorus have been taken into account. Zai et al. used surfactant-aided electrochemical deposition to prepare several layers of ultrathin CoP nanosheets on the surface of NiO_x_ nanotube arrays (Figure 2f) [21]. This heterogeneous structure accelerated mass diffusion, boosted electrical conductivity, scaled down the Gibbs free energy of Co sites to −0.12 eV (Figure 2g), and allowed H_2_O dissociation and hydrogen formation processes to occur at different active sites. Salem and coworkers synthesized flower-like Mn-Ni-Co phosphide catalysts on the NF surface by electrodeposition and a subsequent PH_3_ plasma phosphatization process (Figure 2h) [54]. When the molar ratio of the three elements was 1:1:1, an overpotential of only 14 mV was required to obtain a current density of 10 mA cm^−2^.

However, the stability of TMPs remains far inferior to that of Pt catalysts. During a prolonged electrolysis operation, the active sites of TMPs tend to be corroded and become inactive. Based on the above, various coating protection methods for metal phosphides have been formulated. One of the most representative methods is carbon coating [55,56]. The coated carbon layers not only protect the internal active sites from erosion but also significantly improve overall electrical conductivity. However, while improving catalyst stability, the multilayered carbon shells generated during the carbon coating process tend to cover up the active sites, which means it is difficult to balance activity and stability. Recently, to address the effect of the overcoating of carbon shells on the mass transfer of HER catalysts, etching and defect processes for carbon shells have been widely researched, with the goal of exposing internal metal active sites.

**Figure 3:**
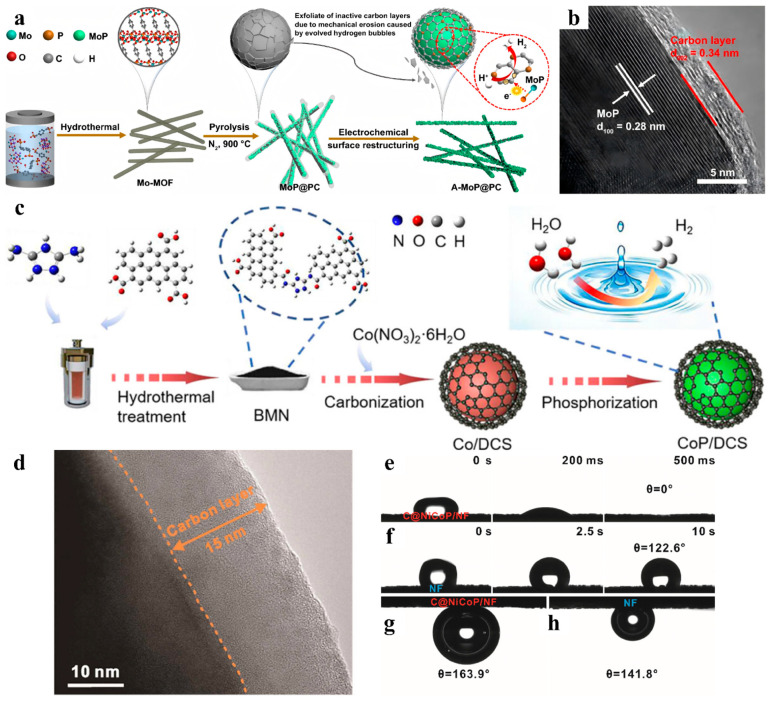
(**a**) Schematic illustration of the synthesis of A-MoP@PC. (**b**) HRTEM image of MoP@PC. Reproduced with permission [57]. Copyright 2021, Springer. (**c**) Schematic of the synthesis process of CoP/DCS. Reproduced with permission [58]. Copyright 2022, Wenzhou University and John Wiley & Sons Australia, Ltd. (**d**) HRTEM image of C@NiCoP/NF. Droplet contact angle comparison of (**e**) C@NiCoP/NF and (**f**) NF. The underwater gas bubble contact angle comparison of (**g**) C@NiCoP/NF and (**h**) NF. Reproduced with permission [59]. Copyright 2025, Elsevier.

Generally, the typical methods for manufacturing defective carbon shells have been pyrolysis and corrosive gas etching. The defects produced by the pyrolysis method mainly depend on the cleavage and release of micromolecular compounds in the carbon precursor. Its disadvantage is that it is difficult to control the number and type of defects. The corrosive gases introduced in the corrosive gas etching method can easily lead to the passivation of the active sites and collapse of the carbon structure. To solve the above issues, Jiang et al. employed a simple electrochemical cycling procedure to remove the inactive carbon layer on the surface and allowed the coated MoP to be tightly bonded to several layers of coated graphene (Figure 3a,b) [57]. Wu and coworkers developed a self-assembly method using modified polycyclic aromatic molecules to encapsulate CoP nanoparticles in defect-rich carbon shells (DCS) [58]. The grafting and removal of the high-activity C-N bonds of aromatic molecules successfully achieved the controllable generation of carbon shell defects, striking a balance between the stability and activity of TMPs (Figure 3c). The experimental results demonstrated that the overpotential (88 mV for 10 mA cm^−2^) and Tafel slope (56 mV dec^−1^) of CoP/DCS catalysts in the alkaline HER reaction were very close to those of the Pt catalysts. Uniform carbon coating on the catalyst surface is another option to balance the activity and stability of TMP [59]. During phosphatization, a 15 nm-thick carbon layer was successfully attached to the leaf-like NiCoP surface (Figure 3d). The existence of the carbon layer serves as a robust safeguard, ensuring the sustained and unwavering stability of the electrode during the reaction process. In addition, the near 0° droplet contact angle (Figure 3e,f) and extremely high underwater bubble contact angle (Figure 3g,h) of C@NiCoP/NF proved that the presence of the carbon layer facilitated the wetting of the electrolyte and the separation of H_2_.

#### 4.1.3. Transition Metal Sulfides

**Figure 4:**
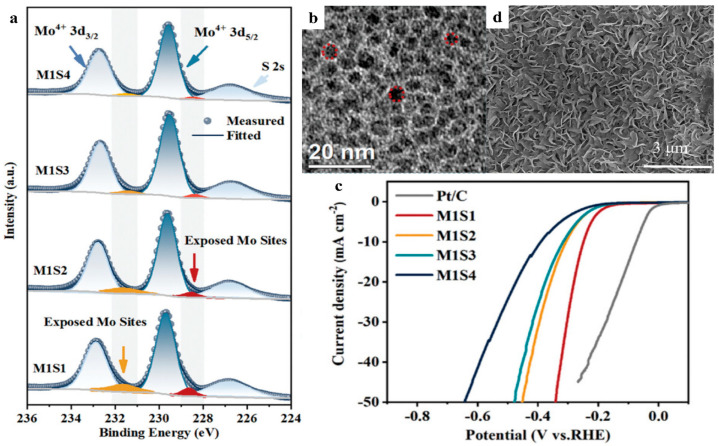
(**a**) XPS spectra of MoS_2_ with different molybdenum–sulfur source ratios. (**b**) TEM image of MoS_2_ with molybdenum–sulfur source ratio of 1:1. (**c**) LSV curves of HERs for different structured MoS_2_ and commercial Pt/C catalysts. Reproduced with permission [60]. Copyright 2023, Wiley-VCH. (**d**) Adsorption–desorption isotherm curve of CN. Insert shows its pore size distribution curve [61]. Copyright 2024, Elsevier.

Adding elements from the oxygen family can enhance the catalytic performance of non-noble metals. Among these, research on metal sulfides is in full swing. MoS_2_ is favored for its ultra- reactive edges and defects. These invaluable edges and defects facilitate the further modification of MoS_2_. In general, the S sites in MoS_2_ demonstrate strong adsorption of H_2_O, while the Mo sites are favorable for m-H adsorption/desorption. As for the edge active sites, Chen et al. created pores in MoS_2_ in situ, creating edge-rich nano islands by adjusting the ratios of S/Mo in hydrothermal reactions (Figure 4b) [60]. When the initial S/Mo ratios were lower than the stoichiometric ratios of the theoretical reaction, abundant undercoordinated Mo atoms existed at the edges of the nano islands (Figure 4a), and the exposure of such Mo active sites led to the rapid increase of HER activity (Figure 4c). The formation of porous, Mo-edge-rich nano islands can be explained by the fact that the limited sulfur source was rapidly depleted during the growth of MoS_2_, and the surplus Mo atoms formed the termination edges on the already constructed MoS_2_ frameworks. During hydrothermal synthesis, MoS_2_ was prone to agglomeration, which resulted in a severe reduction in edge active sites; this phenomenon could be effectively avoided by introducing a nitrogen-containing carbon carrier (CN) [61]. The CN carrier, featuring abundant micropores, facilitates the vertical growth of Cobalt-doped MoS_2_, thereby effectively exposing its active edges (Figure 4d).

**Figure 5:**
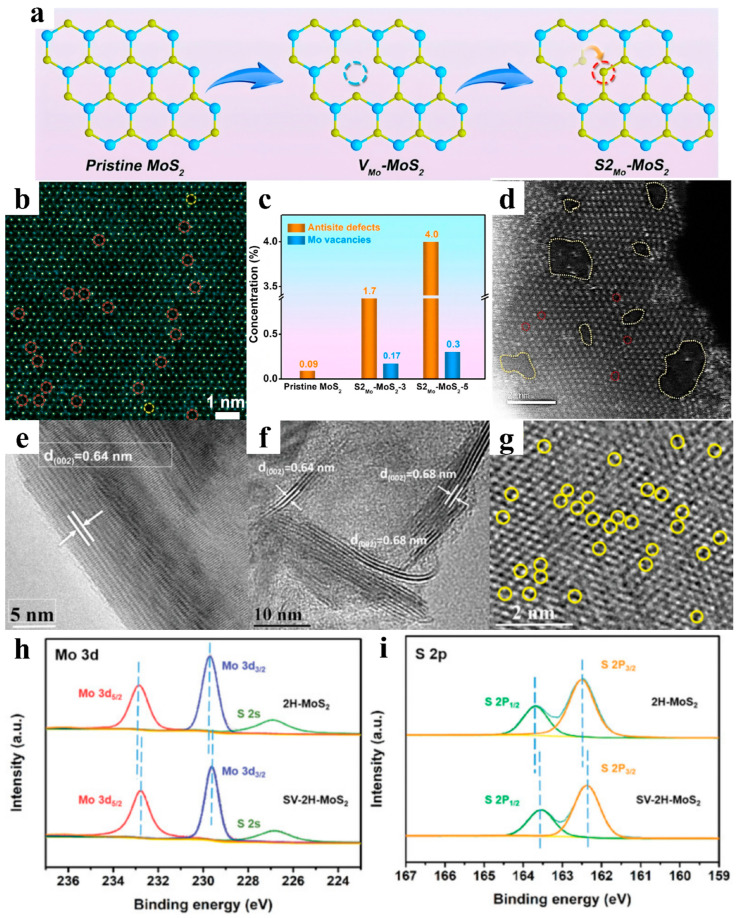
(**a**) Schematic diagram of formation process of MoS_2_ antisite defect. (**b**) Large-region atomic-resolution HAADF-STEM image of S_2_Mo-MoS_2_-5. (**c**) Defect concentration statistics for pristine MoS_2_, S_2_Mo-MoS_2_-3, and S_2_Mo-MoS_2_-5 [62]. Copyright 2023, Nature Publishing Group. (**d**) HAADF-STEM image of defect-rich 1T-2H MoS_2_NS/CFP. Reproduced with permission [66]. Copyright 2022, Elsevier. Crystal lattice spacing for (002) plane of (**e**) 2H-MoS_2_ and (**f**) SV-2H-MoS_2_. (**g**) HAADF-STEM image of SV-2H-MoS_2_. (**h**) High-resolution Mo 3d XPS spectrum and (**i**) high-resolution S 2p XPS spectrum of 2H-MoS_2_ and SV-2H-MoS_2_. Reproduced with permission [67]. Copyright 2023, Wiley-VCH.

When it comes to defects, the common types are point defects, line defects, and surface defects. Point defects are the most common defects in all types, and usually exist in the form of vacancies, antisite defects, nanoscale pinholes, etc. Xu et al. successfully prepared highly efficient HER catalysts with antisite defect structures on the substrate of 2D monolayer MoS_2_ (Figure 5a) by H_2_/Ar atmosphere-assisted calcination [62]. MoS_2_ annealed for 5 min (denoted as S2_Mo_-MoS_2_-5) exhibited a unique structure of two S atoms filling one Mo vacancy (Figure 5b), which possessed an antisite defect concentration of approximately 4.0% (Figure 5c). This kind of catalyst could easily achieve an overpotential of 169 mV for 10 mA cm^−2^ and a Tafel slope of 56 mV dec^−1^ in the HER. The total and partial density of states (DOS), as well as the charge density distribution, showed that the Mo vacancy and antisite defects introduced by the annealing process not only induced the MoS_2_ semiconductor-to-metal transition, which improved electrical conductivity, but also generated a completely different and asymmetric charge density distribution, activating more electron-donating sites for proton adsorption. In recent years, annealing has been widely reported as a favorable method for defect generation in 2D MoS_2_ [63,64,65]. In addition to annealing, other alternative methods can also be employed for the creation of defects. Xie and coworkers synthesized defect-rich 1T-2H MoS_2_ on carbon fiber paper (CFP) through a simple hydrothermal method, and HAADF-STEM characterization (Figure 5d) clearly demonstrated the co-existence of nanoscale pinholes (1–2 nm) and atomic vacancies on the basal plane of this catalyst (indicated by yellow and red dashed lines, respectively) [66]. Gu et al. presented a method of utilizing H_2_O_2_ for electrochemical etching to introduce S vacancies, which was evidenced by the expansion of the (002) planar lattice spacing (Figure 5e,f), the increase of electron density around the Mo and S sites (Figure 5h,i), and HAADF images (Figure 5g) [67]. In addition, using an acidic K_2_Cr_2_O_7_ solution for the chemical etching of KMoS_2_ crystals synthesized by a high temperature solid-state reaction successfully introduced S vacancies [68]. The Mo-Mo bonds retained in the metastable trigonal MoS_2_ (1T‴-MoS_2_) after etching were successfully activated by S vacancies, and the activated metal–metal bonds were able to self-regulate the electronic states and promote proton adsorption by enhancing the S-H bonds. 1T‴-MoS_2_-10.6%, with a large number of S vacancies, was not only three orders of magnitude more electrically conductive than 2H-MoS_2_-VS, but also had higher phase stability in the HER. In electrochemical tests, 1T‴-MoS_2_-10.6%, with one-hour oxidative etching, exhibited an overpotential of 158 mV at 10 mA cm^−2^ and a Tafel slope of 74.5 mV dec^−1^ in acidic media, and the HER current maintained excellent stability in the chronoamperometry (CA) analysis for 24 h without obvious attenuation. As for the introduction of line defects, the control of oxygen concentration during the chemical vapor deposition (CVD) was effective. MoS_2_ was oxidized along the grain boundaries to produce line defects, providing abundant active sites for the HER [69].

**Figure 6:**
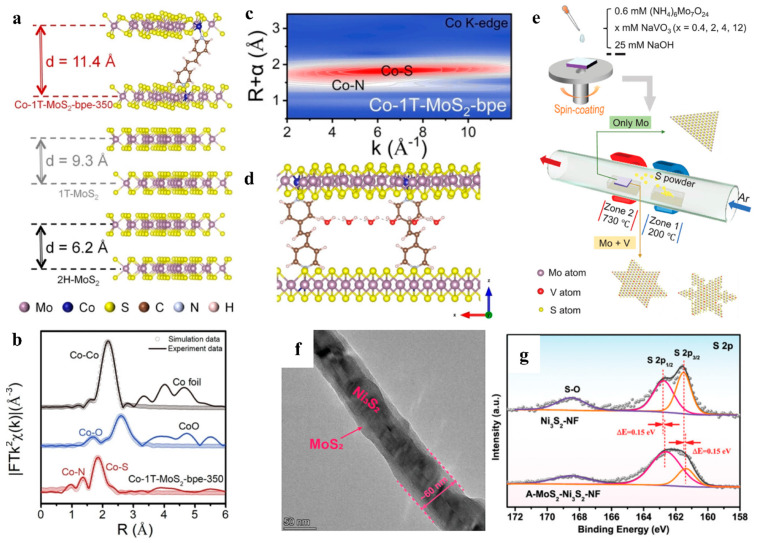
(**a**) Schematic illustration of interlayer spacing of Co-1T-MoS_2_-bpe-350, 1T-MoS_2,_ and 2H-MoS_2_. (**b**) Fourier transformation at R space of Co foil, CoO, and Co-1T-MoS_2_-bpe-350. (**c**) Wavelet transform contour plots of Co foil, CoO, and Co-1T-MoS_2_-bpe-350. (**d**) Schematic of migration of H_2_O molecules in Co-1T-MoS_2_-bpe interlayer in HER-catalyzed reaction. Reproduced with permission [71]. Copyright 2023, Wiley-VCH GmbH. (**e**) Synthesis and characterizations of V-MoS_2_. Reproduced with permission [74]. Copyright 2022, Wiley-VCH. (**f**) TEM image of A-MoS_2_-Ni_3_S_2_. (**g**) S 2p XPS spectra of Ni_3_S_2_-NF and A-MoS_2_-Ni_3_S_2_-NF. Reproduced with permission [76]. Copyright 2023, Wiley-VCH.

In addition to the above improvements for making defects, the HER performance of MoS_2_ can also be boosted by phase engineering, building heterogeneous structures, and regulating interlayer spacing. These aforementioned strategies are usually implemented simultaneously and show excellent synergistic effects [70,71,72]. Yu et al. constructed an interlayer expanded MoS_2_ with a mixture of 1T and 2H phases, which further formed a MoO_2_@E-MoS_2_ heterostructure with MoO_2_ [70]. The introduction of the 1T phase and the interlayer expansion of MoS_2_ improved electrical conductivity, enhanced the adsorption of H_2_O, and exhibited the lowest Gibbs free energy of hydrogen adsorption ( $\Delta G_{H^{*}}$). At the same time, the synergistic effect of heterogeneous structures intensified the above characteristics. With the aid of the 1T-MoS_2_ phase transition and interlayer spacing expansion, Liu and coworkers accomplished the in situ doping of Co atoms on the 1T-MoS_2_ basal plane and the in situ insertion of organic polydentate 1,2-bis(4pyridyl)ethane (bpe) ligand molecules into 1T-MoS_2_ layers by using high temperature sulfurization [71]. The 9.4 Å bpe ligand enlarged the layer spacing to 11.4 Å (Figure 6a,d), which allowed the H_2_O molecules to enter the intermediate layer of the 2D material easily. The doping of Co atoms can adjust the arrangement of S atoms and promote the transition of the semiconductor 2H phase to the metallic 1T phase. Furthermore, Fourier-transformed (FT) k^2^-weighted extended X-ray absorption fine structure (EXAFS) and wavelet transform (WT) contour plots (Figure 6b,c) suggested that the N atoms in the bpe were connected to the cobalt atoms doped in MoS_2_, which demonstrated that both heteroatoms and molecular intercalators were important for the expansion of the layer spacing.

The introduction of other metal or nonmetal active sites may produce unexpected synergistic effects with MoS_2_. For instance, Ni nanoparticles were anchored on MoS_2_ nanosheets by electrodeposition [73]. The Mo sites acted as an adsorption site for H_ad_ and the Ni sites demonstrated a more stable adsorption of OH_ad_, which prevented the recombination of OH_ad_ and H_ad_. This synergistic effect from the Ni/MoS_2_ interface resulted in the rapid departing of the generated small bubbles from the electrode surface, reducing the risk of damage to the electrode by bubbles. Similarly, vanadium (V) atoms were doped on the basal plane of MoS_2_ by CVD to make MoS_2_ a degenerate semiconductor (Figure 6e), and the V dopants synergistically interacted with the S vacancies to change the hydrogen adsorption Gibbs free energy significantly [74]. It was demonstrated that the three V-atom aggregated samples, with a V-doping concentration (CV) of 5.2 at%, had the best catalytic performance, with an overpotential as low as 198 mV at 10 mA cm^−2^ in the 0.5 m H_2_SO_4_ electrolyte. This phenomenon can be explained by the fact that the lower CV leads to a low number of defects, which cannot activate enough adjacent S atoms. However, the higher CV makes the binding strength of the V-Mo to the adsorbed hydrogen excessively strong, making it difficult for H_2_ to escape.

The low conductivity of MoS_2_ is also a major limitation to its catalytic performance. Mravik and coworkers used an ion irradiation method to improve the conductivity of flower-like MoS_2_, in which hydrogen and carbon ions with different energies and fluences were applied [75]. Subsequent electrochemical experiments showed that the flower-like MoS_2_ exhibited the lowest overpotential and the lowest value of Tafel slope after irradiation by high hydrogen ion fluence. Hu et al. grew amorphous MoS_2_ with a highly conductive urchin-like Ni_3_S_2_ skeleton on NF using a simple one-step hydrothermal method. This A-MoS_2_-Ni_3_S_2_-NF core–shell structure (Figure 6f) was demonstrated to have an electron-rich surface by XPS characterization (Figure 6g) [76]. At a current density of 10 mA cm^−2^, this MoS_2_ electrode, attached to highly conductive Ni_3_S_2,_ requires overpotentials of only 95 mV (1.0 m KOH) and 145 mV (0.5 m H_2_SO_4_) in the HER.

**Figure 7:**
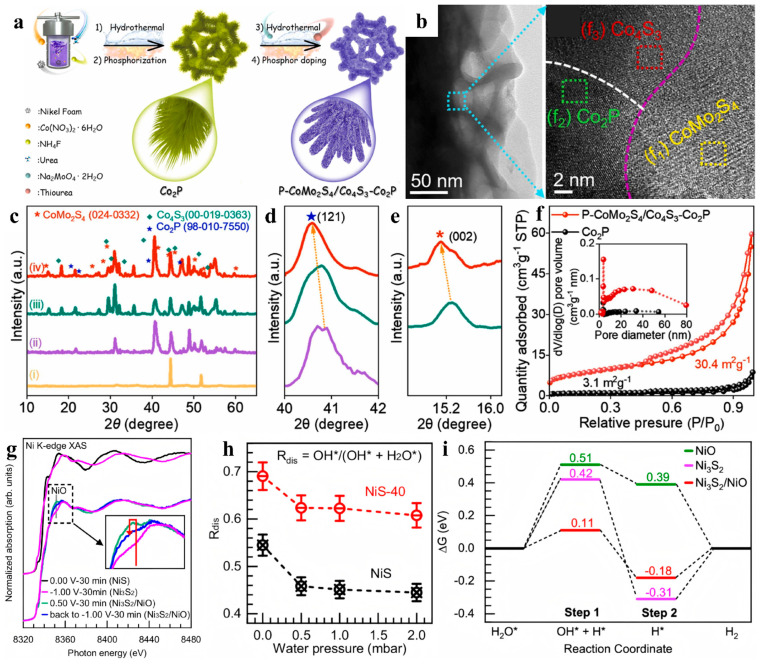
(**a**) Schematic of preparation process of P-CoMo_2_S_4_/Co_4_S_3_-Co_2_P. (**b**) TEM images of P-CoMo_2_S_4_/Co_4_S_3_-Co_2_P structure. (**c**–**e**) XRD patterns of (iv) P-CoMo_2_S_4_/Co_4_S_3_-Co_2_P, (iii) CoMo_2_S_4_/Co_4_S_3_-Co_2_P, (ii) Co_4_S_3_-Co_2_P, and (i) Co_2_P. (**f**) Nitrogen adsorption–desorption isotherms and porous distribution of P-CoMo_2_S_4_/Co_4_S_3_-Co_2_P and Co_2_P materials. Reproduced with permission [83]. Copyright 2023, Elsevier. (**g**) Operando XAS pattern at Ni K-edge of NiS. (**h**) Ratio of water dissociation at different water pressures for NiS-40 and NiS. (**i**) Gibbs free energy diagrams of interfacial Ni-S sites of Ni_3_S_2_/NiO and references. Reproduced with permission [84]. Copyright 2024, Nature Publishing Group.

In addition to MoS_2_, there are many other types of sulfides that exhibit extensive adjustability and good HER performance. Jin et al. achieved precise control of different concentrations of S vacancies in NiS_2_ nanosheets using an Ar plasma etching strategy [77]. The concentration of S vacancies in the nanosheets was proportional to the etching time, and the best m-H adsorption was observed at a vacancy concentration of 5.9%, with an extremely low overpotential (108 mV for 10 mA cm^−2^) and low Tafel slope value (82 mV dec^−1^) in alkaline media. However, the plasma etching method requires complex instruments and high synthesis costs, limiting its widespread application. At present, a series of simple and low-cost defect and edge construction methods represented by microwave-assisted [78] and rapid Joule heating [79,80] technologies have been developed. For instance, microwave-assisted heating enables rapid temperature rise while ensuring more uniform temperature distribution, reducing local overheating or undercooling [78]. The synthesized Ni_3_S_2_/NiO catalyst contains abundant edge sites and in-plane interface sites. Edge sites facilitate binding with OH^−^, promoting surface reconstruction of the catalyst during electrocatalysis; in-plane interface sites enhance proton adsorption capacity. Nonetheless, the microwave-assisted approach for defect or edge site engineering is currently confined primarily to noble metal HER catalysts [81,82], with limited exploration in non-noble metal catalytic systems. Dong et al. developed a P-CoMo_2_S_4_/Co_4_S_3_-Co_2_P heterostructured catalyst grown on three-dimensional nickel foam (Figure 7a) [83]. It exhibited a rich pore structure at the three-phase interface, with P doping (Figure 7b) and crystalline mismatches due to differences in the electronegativities of atoms with multiple different radii (Figure 7c–e). At the same time, it was observed that this heterogeneous structure had a specific surface area of 30.4 m^2^ g^−1^, several times higher than that of a single-component system without a heterogeneous structure (Figure 7f). A large surface area corresponds to more exposure of active sites, which was beneficial for charge transfer and mass diffusion. In alkaline solution, a current density of 10 mA cm^−2^ was achieved with an overpotential of only 54 mV and a Tafel slope of 61 mV dec^−1^. Zhang and coworkers observed the dynamic reorganization of NiS in the HER reaction (Figure 7g) and found that the Ni_3_S_2_/NiO, formed in situ, was the active center [84]. The sample measured at −1.00 V for 40 min (labeled as NiS-40) exhibited a much higher capacity for dissociative adsorption of water than NiS (Figure 7h), allowing the energy barrier of water dissociation to reach 0.11 eV (Figure 7i).

Transition metal selenides and tellurides are also two categories of efficient HER catalysts. Their anions, belonging to Group VIA like those in transition metal sulfides [85], share the same common valence states. They share a similar crystal structure; for instance, MoS_2_, MoSe_2_, and MoTe_2_ all exhibit layered hexagonal structures, with interlayer bonding via van der Waals forces. The decreasing electronegativity of anions (S > Se > Te) enhances electron delocalization, endowing selenides and tellurides with relatively better electrical conductivity [86]. Although tellurides show lower stability, they typically feature higher surface coordination unsaturation, which is prone to generating abundant active sites. Ma and colleagues introduced a Fe-doped NiSe/Ni_3_Se_2_ heterojunction catalyst prepared via a one-step electrodeposition method [87]. Fe^3+^ was introduced through lattice doping, promoting electron transfer from Ni to Fe, reducing the electron density of Ni^2+^, and increasing carrier density and intrinsic conductivity. DFT calculations showed that Se remained the active site for the HER. After Fe doping, the Gibbs free energy of hydrogen adsorption at the Se site decreased from 0.149 eV to −0.051 eV, approaching the ideal value (0 eV), facilitating hydrogen adsorption and desorption. Karthikeyan et al. synthesized carbon-coated bimetallic CoMnTe_2_ nanorods, which exhibited low hydrogen adsorption free energy (0.064 eV) [88]. The d-band center of CoMnTe_2_ was closer to the Fermi level than that of monometallic tellurides, enabling higher charge transfer efficiency. Meanwhile, CoMnTe_2_ showed an overpotential of 136 mV at 10 mA cm^−2^ in alkaline seawater electrolyte, demonstrating its adaptability to seawater electrolysis.

#### 4.1.4. Transition Metal Oxides (TMOs)

Transition metal oxides are versatile and readily available, offering unlimited possibilities for their application in electrocatalytic hydrogen production [89]. Also, transition metal oxides have easily adjustable metal valence states. Different oxidation states of the same transition metal have different catalytic properties. Therefore, the mixture of multiple valence states may show unexpected synergistic effects.

Tungsten-based oxides (WO_3−x_, 0 ≤ x < 3) can form various oxidation states ranging from +6 to 0 due to their complex electronic structure with open d and f shells. Abundant oxygen vacancies provide good electrical conductivity and excellent hydrogen absorption energy for tungsten oxides. These outstanding properties enable WO_3−x_ to exhibit excellent HER activity in acidic media. Yang et al. analyzed the m-H adsorption/desorption capacity of WO_3_ with different crystalline phases using DFT calculations, which showed that the monoclinic phase (m-WO_3_) had a lower Gibbs free energy change than that of the hexagonal phase (h-WO_3_) [90]. Meanwhile, less electron transfer from W to H on the surface of m-WO_3_ (002) suppressed the overlap between the W d_z2_ orbitals and the H_1s_ orbitals, corresponding to a weaker W-H bond than that of h-WO_3_, which facilitated the rapid desorption of the surface-active M-H intermediate. The introduction of reduced graphene oxide (rGO) enhanced the charge transfer of m-WO_3_, and the m-WO_3_/rGO catalyst only needed a 35 mV overpotential to drive a current density of 10 mA cm^−2^ in an acidic electrolyte with a Tafel slope of only 32 mV dec^−1^.

**Figure 8:**
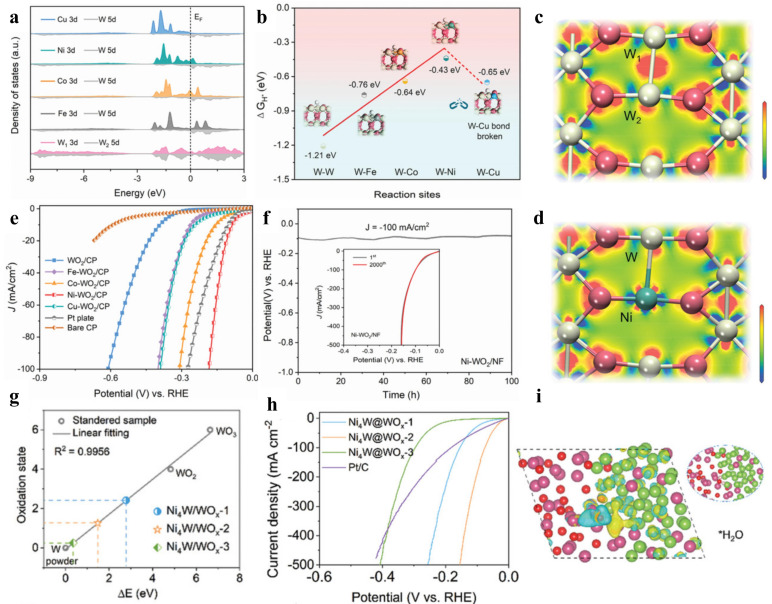
(**a**) DOS plots of W-W bands in WO_2_ and W-M bands in M-WO_2_. (**b**) The hydrogen adsorption Gibbs free energy for different M-W bands in M-WO_2_ (011); M represents W, Fe, Co, Ni, and Cu. The top-view electron density difference of (**c**) WO_2_ (011), and (**d**) Ni-WO_2_ (011), ranging from −0.1 to 0.1 e Å-3. (**e**) LSV curves of WO_2_/CP, M-WO_2_, Pt plate, and bare CP. (**f**) Long-term stability test of Ni-WO_2_/NF. Inset includes the initial and 2000th polarization curves of Ni-WO_2_/NF. Reproduced with permission [91]. Copyright 2022, Wiley-VCH. (**g**) Oxidation states of W in Ni_4_W/WO_x_ with different annealing temperatures. (**h**) Polarization curves of Ni_4_W/WO_x_ and reference. (**i**) Charge density difference of Ni_4_W/WO_x_ during H_2_O adsorption. Reproduced with permission [93]. Copyright 2024, Wiley-VCH.

However, acidic oxides make tungsten-based oxides highly susceptible to dissolution in alkaline media, hindering the alkaline HER. Aiming to improve the activity and stability of tungsten-based oxides in alkaline media, Liu and coworkers introduced the transition metals Fe, Co, Ni, and Cu into WO_2_ for d-d orbital modulation, which revealed a trend of linear decoupling between the W-5d orbitals and the M-3d orbitals when the metal heteroatom replaced W to form the W-M bond (Figure 8a) [91]. Among them, the W-Ni bond formed by Ni doping redistributed the internal charges of WO_2_ and significantly diminished the electron density around the W sites (Figure 8c,d), resulting in a Gibbs free energy change of −0.44 eV for the W-Ni sites (Figure 8b). Ni-WO_2_/NF achieved an overpotential of 41 mV (at 10 mA cm^−2^) in 1 m KOH electrolyte and successfully worked stably for more than 100 h at a current density of 100 mA cm^−2^ (Figure 8e,f). Chen et al. synthesized a metallic heterostructured W/WO_2_ HER catalyst featuring the co-existence of two tungsten-based phases, which exhibited impressive activity and structural robustness in alkaline media [92]. WO_2_, as a relatively special tungsten oxide species that combines both metallic and oxidative properties and has oxygen vacancies and metallic properties, accelerated the adsorption and cleavage of H_2_O and promoted the formation of hydrogen tungsten bronze (H_x_WO_y_) intermediates. In addition, the introduction of zero-valence W (W^0^) sites circumvented the slow hydrogen desorption kinetics of H_x_WO_y_ intermediates and promoted the rapid cycling of the active sites. The high robustness of W/WO_2_ in high-pH media is attributed to the following factors: (I) The co-existence of metals and oxides in W/WO_2_ heterostructures resulted in sound oxidation resistance; (II) Intense chemical and electronic interactions occurred between W and WO_2_ components; (III) The introduction of a carbon source during the preparation of W/WO_2_ catalysts by a pyrolytic reduction method generated surface carbon layers that prevented the corrosion of alkaline solutions on the electrodes. The rational design of NiW/WO_2_ heterostructures, which effectively regulates the oxidation states of tungsten, significantly enhances alkaline HER activity [93]. According to the X-ray absorption fine structure (XAFS) fitting, the oxidation state of W in the sample of Ni_4_W/WO_x−2_ obtained by annealing at 600 °C was +1.48 (Figure 8g), which corresponds to optimal HER activity (Figure 8h). The oxidation state of W gradually decreased during the HER process (Figure 8i), revealing its key role as an H_2_O dissociation site.

**Figure 9:**
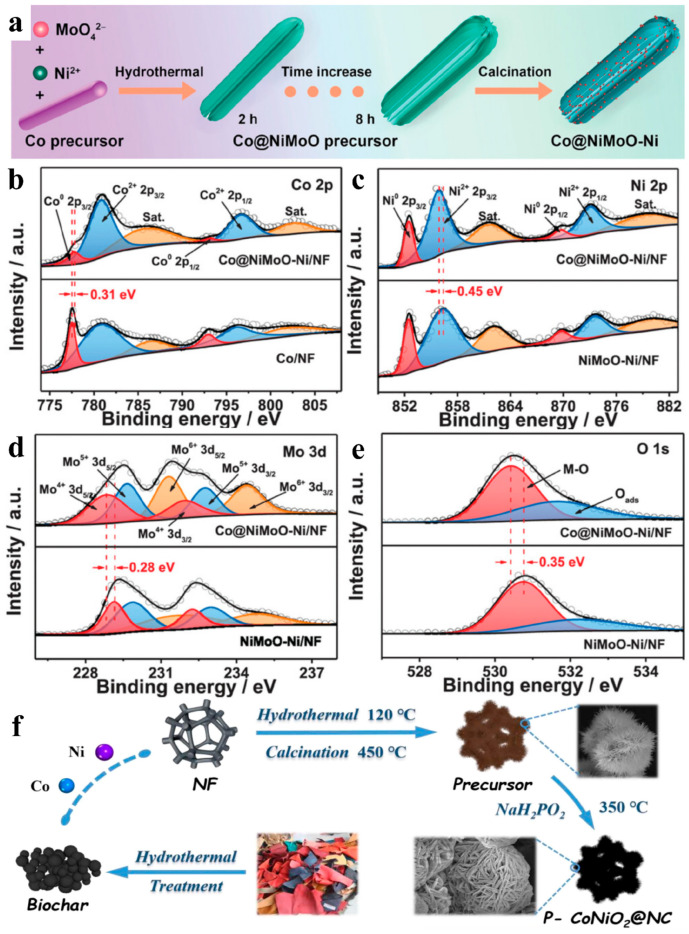
(**a**) Schematic illustration of the synthetic process of Co@NiMoO-Ni. XPS spectra of (**b**) Co 2p, (**c**) Ni 2p, (**d**) Mo 3d, and (**e**) O 1s for Co@NiMoO-Ni/NF, NiMoO-Ni/NF, and Co/NF [3]. Copyright 2023, Wiley-VCH. (**f**) Schematic illustration of the synthetic process of P-CoNiO_2_@NC. Reproduced with permission [94]. Copyright 2023, Elsevier.

Apart from tungsten-based oxides, many diverse non-noble metal-based oxides exhibit extraordinary catalytic activity during HER. Wu et al. synthesized Ni nanoparticle-decorated NiMoO nanosheets based on Co nanowires (Co@NiMoO-Ni/NF) through hydrothermal and calcination processes (Figure 9a) [3]. Its extremely low overpotential (18 mV for 10 mA cm^−2^) and Tafel slope (27 mV dec^−1^) was attributed to the co-existence of high-valent Ni^2+^ and Mo^6+^ and low-valent elements Ni^0^, Mo^4+^, and Mo^5+^. Among them, Ni^2^+ effectively weakened the H-O bond of adsorbed H_2_O molecules, Ni^0^ provided H adsorption sites, and low-valent Mo sites promoted H_2_ desorption. Its high electrical conductivity was due to the strong interaction between Co and NiMoO-Ni. Comparing the binding energy of Co 2p in Co@NiMoO-Ni/NF with NiMoO-Ni/NF and Co/NF (Figure 9b–e) indicated that the electrons were transferred from Co to NiMoO-Ni, which formed two oppositely charged regions and facilitated the charge transfer. Zhao and coworkers introduced N-doped carbon substrate (NC) and P into TMOs to form a quintessential P-CoNiO_2_@NC catalyst (Figure 9f) [94]. The NC originated from waste-derived carbon substrates (WDCS) that were obtained by hydrothermal pretreatment of waste cotton textile (WCT). Both experiments and theoretical calculations showed that the WDCS and P doping promoted the HER catalytic activity of a wide variety of dual 3d TMOs such as CoNiO_2_, CoFe_2_O_4_, NiFe_2_O_4_, and so on. Analogously, potato starch carbon aerogel enriched with amorphous CoO_x_ (CoO_x_/PSCA) is another typical case of a composite of TMOs with carbon materials [95]. The CoO_x_/PSCA exhibited a three-dimensional mesh structure with copious pores, which provided plentiful mass electron transfer channels for the HER reaction, and this structure could remain morphologically stable during the 10 h i-t curve test. Shao’s group transformed the insulating scheelite-structured BaMoO_4_ into metallic perovskite-structured BaMoO_3_ via a thermal-reduction-induced phase transition [96]. In 1 m KOH electrolyte, BaMoO_3_ achieved an overpotential of 336 mV at a current density of 10 mA cm^−2^, significantly lower than the 561 mV exhibited by BaMoO_4_, with an approximately one-order-of-magnitude enhancement in intrinsic activity. This performance improvement was attributed to the stable Mo^4+^ valence state in BaMoO_3_, increased surface oxygen vacancy concentration, and a conductivity enhancement exceeding seven orders of magnitude from 3.4 × 10^−6^ S/cm to 1.4 × 10^2^ S/cm.

### 4.2. MOFs and Their Derivatives

MOFs are porous crystalline materials composed of metal ions (Cu^2+^, Zn^2+^, Cr^3+^, Co^2+^, Fe^3+^, Ni^2+^, etc.) and organic ligands. They have diverse species, highly ordered pores, low crystal density, high specific surface area, and good tunability [97,98]. However, MOFs tend to exhibit low stability and poor electrical conductivity, hindering their straightforward application in electrocatalysis [99]. Multiple MOF-based derivatives have been developed to utilize the advantages of MOFs and avoid their deficiencies, which has become an important direction for studying the structure–activity relationships of HER catalysts [100].

First, slight modification of MOFs ensured that their basic framework structures remained unchanged, which could be roughly categorized into guest/MOFs and MOF/substrates. For guest/MOFs, Velayutham and coworkers used a hydrothermal method to grow molybdenum disulfide in situ on the Fe-MOF skeleton [101]. When the hydrothermal time was 6 h, Fe-MOF@MoS_2_-6h exhibited a heterogeneous interface structure where nanorods and nanosheets co-existed. The total DOS of Fe-MOF samples before and after MoS_2_ deposition was calculated and analyzed by first principle theoretical calculation. The total DOS showed a crossing with the Fermi energy level, which indicated that Fe-MOF@MoS_2_ had intrinsic metal conductivity.

When it comes to MOF/substrates, Gugtapeh and Rezaei electrodeposited NiCo-MOF on a pyramid-like NiSb surface [102]. Through comparison with the structure of Ni-MOF and Co-MOF, it can be found that NiCo-MOF has outstanding porosity. The porosity reached the maximum value when the Ni/Co ratio was 8:3, which was favorable for electrolyte absorption.

**Figure 10:**
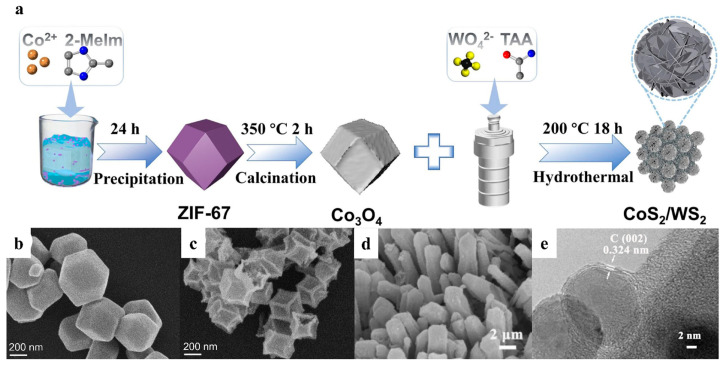
(**a**) Schematic illustration of the preparation process for CoS_2_/WS_2_. SEM images of (**b**) pure ZIF-67 and (**c**) Co_3_O_4_ skeleton. Reproduced with permission [103]. Copyright 2023, Elsevier. (**d**) FE-SEM and (**e**) HRTEM images of MOF-derived CoNi@CNC. Reproduced with permission [105]. Copyright 2024, Elsevier.

In addition, pyrolysis using MOFs as precursors to obtain various derivatives is also a common method to obtain efficient HER catalysts, and MOF precursors can provide high-quality skeletons for their derivatives. Yao et al. prepared CoS_2_/WS_2_ composites by calcination and a one-pot hydrothermal method by using Co-MOF (ZIF-67) as the precursor. ZIF-67 was converted to Co_3_O_4_ during the first step of the calcination process (Figure 10a) [103]. SEM analysis showed that Co_3_O_4_ generated by pyrolysis at 350 °C retained the original dodecahedral framework of ZIF-67 (Figure 10b,c), which featured a unique cavity structure and promoted the loading and growth of WS_2_. Do and coworkers used the hollow Ni-MOF to successfully prepare H-Ni/NiO/C HER catalysts by a two-step process of carbonization in an N_2_ atmosphere and high-temperature oxidation in the air [104]. The carbonization step embedded Ni atoms in the hollow carbon skeleton, and the oxidation process oxidized some of the Ni atoms into NiO phases. H-Ni/NiO/C exhibited a lower overpotential (87 mV for 10 mA cm^−2^) and Tafel slope (91.7 mV dec^−1^) compared to non-hollow NH-Ni/NiO/C, H-Ni/C, and H-NiO/C. This was due to the accelerated breaking of O-H bonds, efficient recombination of adsorbed hydrogen, and rapid transfer of electrons between the metal and metal oxide caused by the hollow structure. Chen and coworkers fully combined the advantages of MOF-74 and ZIF-67 to synthesize MOF derivatives (labeled as CoNi@CNC) with both conductivity and stability under pyrolytic conditions [105]. The annealing process at 500 °C did not change the nanoneedle array structure of the precursor (Figure 10d) and formed an ultrathin carbon shell with a protective effect (Figure 10e), which balanced stability and charge transfer activity.

**Figure 11:**
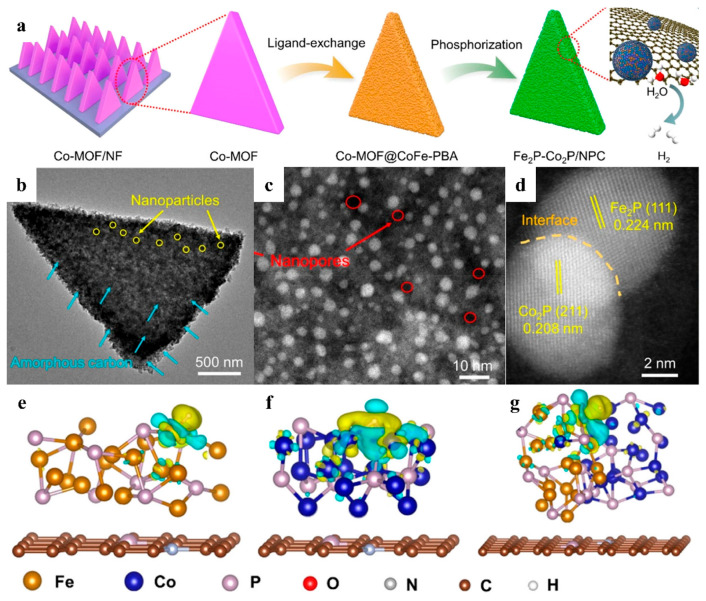
(**a**) Schematic illustration of the synthetic procedure of Fe_2_P-Co_2_P/NPC. (**b**) TEM and (**c**,**d**) HAADF-STEM images of Fe_2_P-Co_2_P/NPC catalyst. Electron density difference in (**e**) Fe_2_P/NPC, (**f**) Co_2_P/NPC, and (**g**) Fe_2_P-Co_2_P/NPC systems, respectively. Reproduced with permission [108]. Copyright 2023, American Chemical Society.

Simultaneously, MOF precursors can also provide their derivatives with abundant sources of various elements. Wang et al. used ZIF-67 grown on the surface of Zn-MOF (ZIF-8) arrays as a precursor, which was converted into Co-embedded N-doped carbon nanotube hybridized arrays (Co-NCNT/NF) by an annealing process [106]. The 2 μmthick ZIF-67, wrapped on the surface of ZIF-8, provided a rich source of carbon, nitrogen, and cobalt for the generation of Co-N-C sites. Ghising and coworkers obtained MOF-derived vanadium and nitrogen co-doped bimetallic selenides (V,N-Co/Fe-Se/C@NF) by applying CVD selenization to V-doped CoFe bimetallic MOFs (V-CoFe-MOF@NF) [107]. V-CoFe-MOF provided the metal elements required for the HER, where Co^2+^ and Fe^3+^ were the active sites, and the lattice defects and atomic distortions induced by the co-doping of vanadium and nitrogen increased the number of these active sites and also enhanced electrical conductivity. Zhang et al. converted Co-MOF into Co-MOF@CoFe-Prussian blue analogue (Co-MOF@CoFe-PBA) using a ligand exchange strategy and prepared Fe_2_P-Co_2_P/NPC catalysts using a subsequent phosphorylation treatment (Figure 11a) [108]. Characterized by TEM and HAADF-STEM, ultrafine Fe_2_P and Co_2_P nanoparticles were uniformly anchored on N and P co-doped porous carbon nanosheets (Figure 11b–d). DFT calculations demonstrated that the transfer of electrons in the heterogeneous interface was oriented from Fe and Co to P (Figure 11e–g), and such interfacial Co-P-Fe bridging lowered the HER energy barrier.

### 4.3. Alloys and Intermetallic Compounds (IMCs)

**Figure 12:**
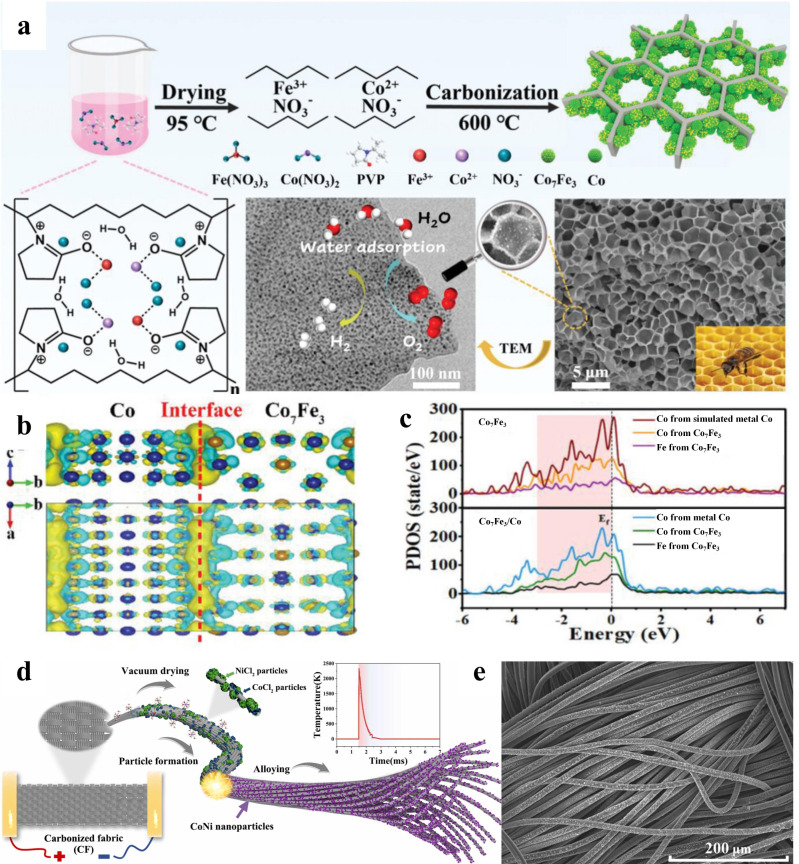
(**a**) A schematic illustration of the synthetic procedure of Co_7_Fe_3_/Co. (**b**) Differential interface charge density (the yellow areas represent charge accumulation and the blue areas represent charge depletion). (**c**) The partial density of states (PDOS) of surface models (Fermi level is denoted by dashed lines). Reproduced with permission [113]. Copyright 2023, Wiley-VCH. (**d**) A schematic of UJH synthesis of Co-Ni@CC, with an inset showing the temperature variation during discharge. (**e**) SEM image of Co-Ni@CC. Reproduced with permission [114]. Copyright 2025, Elsevier.

Alloying is an effective approach to enhance the HER activity of catalysts. The combination of different metals can provide a variety of active sites to adapt to the multi-step reaction of the HER as well as the synergistic effect by adjusting the electronic structure between the metals [109,110,111]. However, pure-phase bimetallic or trimetallic alloys often fail to achieve ideal H adsorption/desorption, while the coupling of alloys with other metallic or nonmetallic substrate brings unexpected interfacial or synergistic effects, which greatly improves the activity and stability of alloys for HER applications. For example, Kumar et al. percolated NiCu alloy onto Co nanosheets to form NiCu/Co alloy catalysts [112]. Due to the synergistic interaction between the three metals, the Cu sites were activated, resulting in a minimum $\Delta G_{H^{*}}$ of 0.02 eV for this catalyst. In the alkaline electrolyte, only 86 mV of overpotential was required to reach a current density of 10 mA cm^−2^ and the Tafel slope was 42 mV dec^−1^. Similarly, Wang et al. co-coupled the Co_7_Fe_3_ alloy and metallic Co onto three-dimensional, honeycomb-like graphitic carbon to form Co_7_Fe_3_/Co heterojunctions (Figure 12a) [113]. The wettability of the sample was studied by measuring the contact angle. The results showed that the contact angle of Co_7_Fe_3_/Co-600 was 8.58°, significantly lower than that of other samples, indicating good hydrophilicity, which corresponds to stronger reactant adsorption and faster bubble desorption. DFT demonstrated that the addition of Co rearranged the electrons of Co_7_Fe_3_, promoting the formation of electron-rich states at the interface (Figure 12b) and electronically delocalized states at the Co and Fe sites (Figure 12c). Yang and coworkers synthesized CoNi nanoalloy catalysts on carbon cloth (Co-Ni@CC) in milliseconds using an ultrafast Joule heating (UJH) technique [114]. The transient high temperature of 2000 K allowed favorable dispersion and chemical stabilization of the Co-Ni alloy on the carbon cloth (Figure 12d,e), substantially augmenting the exposure of active sites and thereby optimizing the adsorption/desorption kinetics of intermediates.

Yu and colleagues prepared a porous NiW bimetallic alloy via a dynamic H_2_ bubble template (DHBT) strategy, with an electrochemically active surface area (ECSA) of 97.6 cm^2^, significantly larger than that of monometallic Raney Ni (4.7 cm^2^) and Ni Mesh (1.6 cm^2^) [115]. The porous network structure corresponding to the large ECSA shortens the diffusion pathways of OH^−^ and H_2_O molecules in the electrolyte to the active sites while accelerating the escape of H_2_ bubbles. Experiments have observed that the H_2_ bubbles generated on the surface of the porous NiW alloy are smaller and released more rapidly, avoiding the clogging of active sites caused by bubble aggregation. Meanwhile, the large active surface area enables close contact between NiW particles, forming a continuous electron conduction network, further accelerating the reaction kinetics. This achieves overpotentials of 198 and 264 mV at industrial-level current densities of 500 and 1000 mA cm^−2^, respectively.

**Figure 13:**
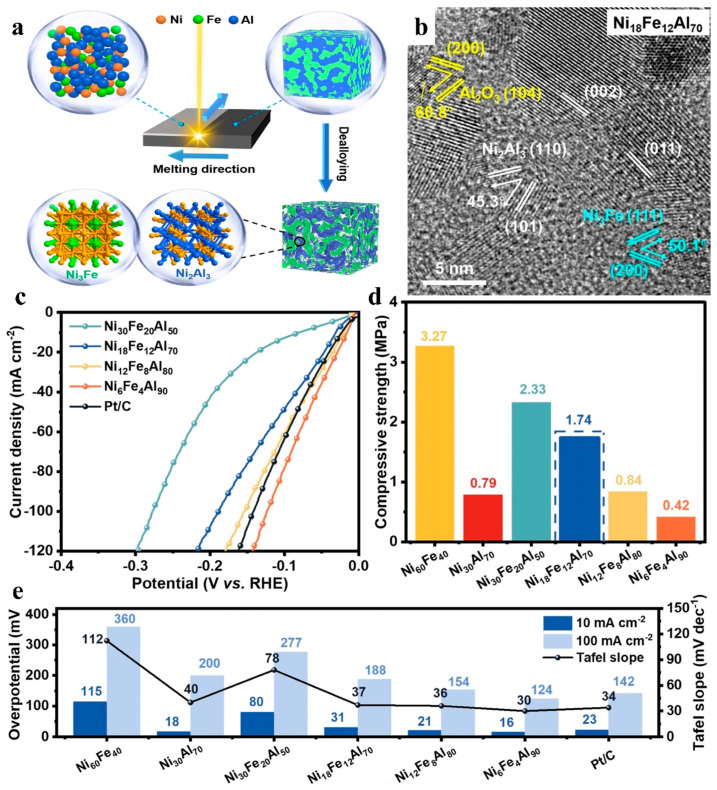
(**a**) Schematic illustration of the synthetic procedure of NiFeAl/NF. (**b**) HRTEM image of Ni_18_Fe_12_Al_70_. (**c**) LSV curves of Ni_30_Fe_20_Al_50_, Ni_18_Fe_12_Al_70_, Ni_12_Fe_8_Al_80_, and Ni_6_Fe_4_Al_90_ during HER process. (**d**) Catalyst compressive strength. (**e**) Overpotential and Tafel slope comparison for different catalysts. Reproduced with permission [117]. Copyright 2023, Wiley-VCH.

Similar to alloys, intermetallic compounds are also combinations of different metals, where multiple metals provide rich and diverse reactive sites. Their electronic structures can be tuned through ligands, geometrical effects, and strain effects [116]. However, unlike the alloys discussed above, intermetallic compounds are usually bonded by both ionic and covalent bonds, and the proportions of these two types of bonds vary from compound to compound. The combination of ionic and covalent bonds endows intermetallic compounds with exceptional properties, resulting not only in a strict crystal structure but also good stability for catalytic processes. Liu and coworkers prepared a nanoporous NiFeAl/NF electrode using a laser direct writing technique and dealloying process (Figure 13a) [117]. The electrode mainly consisted of a Ni_3_Fe alloy phase and Ni_2_Al_3_ intermetallic compound phase (Figure 13b), in which Ni_2_Al_3_ was the main active site of the HER. The LSV tests showed that with the increase of Al content, HER activity increased correspondingly, which was evidenced by a gradual decrease in the overpotential (Figure 13c,e). However, when the Al content exceeded 70%, the decrease in overpotential was extremely insignificant, implying that above this threshold, Al was completely dealloyed. Meanwhile, electronic universal testing was employed to characterize the compression resistance of all the samples with different Al contents. The results demonstrated that Ni_6_Fe_4_Al_90_, which was the most active catalyst, had the lowest compressive strength of 0.42 MPa (Figure 13d), although it had the lowest overpotential (16 mV for 10 mA cm^−2^) and Tafel slope (30 mV dec^−1^). In contrast, Ni_18_Fe_12_Al_70_ had a well-balanced combination of active (31 mV for 10 mA cm^−2^) and mechanical properties (σ_bc_ = 1.74 MPa), and its potential could be stable for 100 h at current densities of 100 mA cm^−2^ and 400 mA cm^−2^. Similar to the above, Zhou et al. accomplished the in situ synthesis of FeNiZn alloys and FeNi_3_ intermetallic compound heterostructures on three-dimensional NiFe foam by employing Zn plating, annealing, and etching processes [118]. FeNiZn/FeNi_3_@NiFe catalysts had high-quality porous and intercrossing heterostructures, realizing the strong synergistic effects of alloys and intermetallic compounds. The synergistic effects of the heterostructures promoted the transfer of electrons from FeNi_3_ to FeNiZn, which made the catalyst obtain a suitable d-band energy level to facilitate the adsorption and desorption steps during the water splitting process.

### 4.4. High-Entropy Alloys and High-Entropy Oxides

In the recent past, a new class of materials known as high-entropy alloys (HEAs) have been increasingly applied in the field of HERs. HEAs can be defined as a series of single-phase alloys composed of five or more metallic elements in approximately equal proportions [109,119]. The tunable arrangement of the multiple elements produces unexpected synergies, and the high phase stability of HEAs significantly enhances their corrosion resistance in acidic or alkaline media [120]. Tong’s group developed a non-noble metal FeCoNiMnZn high-entropy alloy (HEA) nanocatalyst supported on carbon nanotubes (CNTs) [121]. The samples, synthesized via a fast-moving bed heating process, underwent rapid heating and cooling, exhibiting excellent anti-particle aggregation properties. Such nanoscale, uniformly dispersed HEAs enabled the maximum utilization of synergistic effects among various metals. However, the application of HEAs in HERs is still in its infancy due to the complexity of the synthesis and characterization of HEAs, as well as the difficulty in distinguishing active sites because of the presence of multiple elements. Simultaneously, the elements in HEAs often involve noble metals, and HEAs composed entirely of non-precious metals are rare, which means that there is still a long way to go for non-noble, metal-based HEA catalysts.

High-entropy oxides (HEOs) evolved from the concept of high-entropy alloys, referring to the formation of unique structures by dissolving more than five metallic elements in a single lattice [122]. The disordered distribution and synergistic effects of multiple elements can induce lattice distortion, create oxygen vacancies, suppress segregation, and provide more active sites [123]. Kang et al. synthesized spinel-structured HEO/NiFeCuMoMn HEA multiphase catalysts via in situ electrodeposition, which exhibited excellent catalytic activity and stability in a seawater HER [124]. The superior performance originates from the synergistic effect of multiple elements, the effective adsorption of H_2_O and promotion of H^*^ desorption by spinel-structured HEOs, and the corrosion resistance of HEA itself. Meanwhile, the decrease in overpotential from 371 mV to 343 mV during the seawater HER stability test demonstrates its unique autocatalytic ability.

### 4.5. Composite and Heterostructured Catalysts

Heterostructured catalysts are composite catalysts formed by the interfacial bonding of two or more different materials [125]. Although some heterostructured catalysts have been involved in previous classifications, the synergy between heterostructures formed by compounds from different classifications remains fascinating. In this section, we will place greater emphasis on analyzing research progress in DFT calculations of interfacial charge distribution, explaining the causes of performance enhancement in heterostructured catalysts from the perspective of electron interactions. DFT calculation was employed to explore the interfacial electron interaction mechanism of the MoO_2_/Ni_3_S_2_ heterostructured catalyst for an alkaline HER [126]. Electrons flowed from Ni_3_S_2_, which has a lower work function, to MoO_2_, which has a higher work function, leading to charge accumulation at the O atoms of MoO_2_ and charge depletion at the Ni atoms of Ni_3_S_2_. This formed an interfacial built-in electric field that accelerated electron transport. The energy barrier for water dissociation was reduced to 1.17 eV, corresponding to an experimental Tafel slope of 85.2 mV dec^−1^, which was lower than that of either single-phase catalyst.

For the complex Te-MoTe_2_-MoS_2_/ZnO heterostructure catalyst, the DOS overlap at the interface was enhanced, indicating an improved electron transfer efficiency across the interface [127]. Particularly, the low bandgap characteristic of MoTe_2_ promoted the rapid transfer of carriers. DFT calculations have become a necessary and persuasive approach for analyzing key parameters such as interfacial charge transfer and adsorption energy barriers at active sites in heterostructures [128,129]. Being widely applied in an increasing number of studies, they provide valuable guidance for the optimization of HER catalysts.

### 4.6. Carbon-Based Electrocatalysts

**Figure 14:**
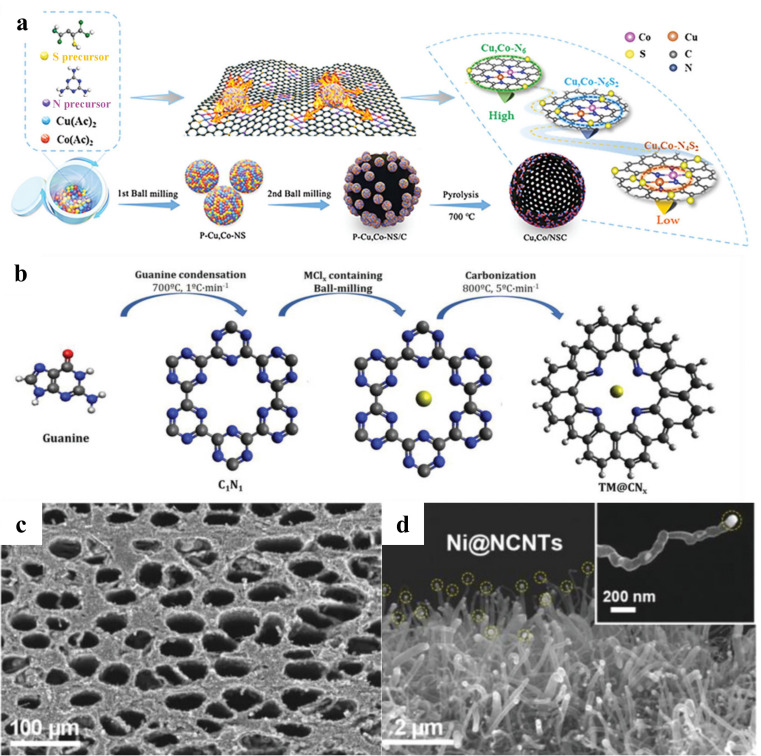
(**a**) Schematic illustration of the procedure to synthesize Cu and Co DACs. Reproduced with permission [134]. Copyright 2023, Wiley-VC. (**b**) Schematic illustration of the procedure to synthesize TM@CN_x_. Reproduced with permission [135]. Copyright 2023, Wiley-VCH. (**c**,**d**) SEM images of Ni@NCW. Reproduced with permission [138]. Copyright 2023, Wiley-VCH.

Alongside forming compounds with transition metals or acting as an active site for doping, carbon can also be used as a support to provide anchoring points for HER catalysts and significantly increase electrical conductivity and stability. At present, relatively well-established carbon matrices mainly include carbon cloth (CC) [130,131], carbon fiber paper [50,66,132], graphene [133], and carbon black [134], which can be purchased directly as chemicals or pre-synthesized very conveniently. The introduction of carbon supports facilitates the synthesis of catalysts. For example, the pre-acidification of the CC substrate ensured its attraction to Co^2+^, which anchored double-shelled hollow CoP nanoparticles on CC, and a uniform loading of Co-based leaf-like nanosheet arrays was achieved, which was crucial for the formation of highly efficient HER catalysts [130]. When synthesizing Cu and Co dual-atom catalysts (Figure 14a), the wet ball milling process on carbon black supports ensured a uniform arrangement of the metal precursor atoms, which facilitated the appearance of single atoms during the high-temperature carbonization process [134].

The addition of N to the carbon substrates can further enhance the HER performance of the catalysts. Quílez-Bermejo et al. prepared the C_1_N_1_ precursor using guanine as the raw material [135]. The C_1_N_1_ had an internal cavity with a diameter of approximately 0.6 nm based on four N atoms (Figure 14b). The transition metals were anchored in the cavity of C_1_N_1_ with N atoms via transition metal–N_4_ (TM-N_4_) bonds. The TM@CN_x_ catalysts were formed after a subsequent pyrolysis step, where x < 1. Among these, Co@CN_x_ with the introduced Co nanoclusters had the highest HER activity, showing an EHER of −0.27 V in alkaline solution. The utilization of N-doped carbon (NC) as a substrate can also improve electrical conductivity and inhibit the agglomeration of nanoparticles [136,137].

Biomass carbon is also a very trendy carbon-based material. By using biomass as a precursor for carbon support materials, it effectively utilizes carbonaceous waste that may have been buried or burned in the past, reduces the degradation pressure on the natural environment, and is inexpensive and easy to obtain. Wang et al. loaded Fe/FeP nanospheres on Ginkgo-leaf-derived carbon by graphitization and phosphating processes, and researched the effect of different Fe contents on the HER properties [139]. The d-band centers of the prepared Fe&FeP@gl-C shifted away from the Fermi level, which was attributed to the synergistic effect between Fe and FeP, and the Fe-P-C sites acted as the main active centers, accelerating the Volmer and Heyrovsky steps, exhibiting a low Tafel slope of 70.37 mV dec^−1^ in a 0.5 m H_2_SO_4_ electrolyte. Li and coworkers obtained carbonized wood (CW) by carbonizing natural beech wood pieces at 900 °C [138]. Subsequently, Ni atoms were encapsulated in N-doped carbon-based frameworks by electrochemical deposition and annealing processes, forming a three-dimensional hierarchical porous integrated electrode structure. During the synthesis process, the addition of melamine led to the formation of a large number of carbon nanotubes on the surface and inside the pores of carbonized wood (Figure 14d). The Ni atoms encapsulated in the nanotubes lose their catalytic activity due to aggregation (Figure 14c). This hierarchical wood-derived carbon-based electrode structure not only had a low overpotential (158 mV 10 mA cm^−2^) but also achieved an industrial-level current of 4.0 A at a low battery voltage of 2.43 V in an anion exchange membrane water electrolysis (AEMWE) electrolyzer.

## 5. Conclusions and Outlooks

In summary, we focus on the cathodic hydrogen evolution reaction during the electrolysis of water and discuss the latest advances in non-noble metal HER catalysts in recent years. We briefly introduce the superiority of hydrogen production via water electrolysis, the basic fundamentals of the HER process, and the performance evaluation parameters of HER catalysts. The various types of non-noble metal HER catalysts are grouped and summarized, and the performance comparison of the non-noble metal HER catalysts detailed in this review is shown in Table 1. Transition metal compounds, MOFs and their derivatives, alloys and intermetallic compounds, as well as carbon support materials, all exhibit excellent prospects. Using advanced characterization technology, performance testing methods, and rigorous theoretical calculations, researchers are gradually expanding our understanding of the HER process of water electrolysis. HER is a process of hydrogen adsorption and desorption, so high activity of the HER depends on a moderate interaction between the active sites and hydrogen. Modulation of the electronic structure of the catalyst is often an effective way to enhance HER activity. The introduction of substrates, formation of alloys, doping of heteroatoms, and construction of heterostructures are all effective methods to modulate the electronic structure.

With completely new catalyst structures emerging, such as MOFs, HEAs, or MXenes, there is likely to be a boom in HER research. This means that advanced synthesis techniques can provide HER catalysts with better morphological structures or special properties. These properties either expose more active sites or modulate the electronic structure, and high-resolution electron microscopy images and spectra can offer a more plausible explanation for their good performance. The emergence of high-quality HER catalysts requires highly sophisticated synthesis techniques and relies on well-established operando dynamic characterization methods for the interpretation of the reaction process. Furthermore, in situ characterization techniques impose increasingly higher requirements on the spatial and temporal resolution of instruments [140]. Enhancing spatial resolution is needed to progress from the spectral acquisition of specific elements to comprehensive mastery of all contained elements. Meanwhile, increasing the probe scanning speed can not only capture short-lived intermediates [141] but also track dynamic behaviors such as catalyst reconstruction, structural evolution, and bubble motion [142,143]. The current means of characterization in the research process over-focuses on diversity and comprehensiveness, while often neglecting accuracy. At the same time, the commonly used in situ characterization techniques do not exactly replicate the real reaction environment, which makes the findings much less convincing. The solution to this problem depends on the development of operando characterization techniques.

In addition to the refinement of synthesis and characterization techniques, the unification of catalytic performance evaluation standards is also an increasingly critical issue. For example, the catalyst loading per unit electrode area has a non-negligible effect on the evaluation of catalytic performance, and this non-linear influence often poses an obstacle to the comparison of performance between different catalysts. Furthermore, the widespread use of substrate materials such as nickel foam, carbon cloth, titanium mesh, etc., makes catalyst loading even more difficult to calibrate. In addition, the arbitrariness in the selection of the DC potential during the EIS test, the selection of the current density in the LSV curve, and the range of the data during the Tafel slope fitting present problems. The above problems objectively made it more confusing to compare the performance of catalysts reported in different works. Therefore, at the comparison stage of performance, there should be more universal standards to follow and clearer labeling for easy reference.

The purpose of researching HER electrocatalysts is to achieve stable and efficient H_2_ production on an industrial scale. Therefore, it is necessary to develop schemes for large-scale synthesis of high-efficiency catalysts, and the catalytic materials should possess high thermal/chemical stability to meet the practical application requirements of long-term and high-load operation [144]. Seawater-based HER is also an important approach for reducing costs and achieving industrial hydrogen production. However, Ca^2+^ and Mg^2+^ ions in seawater readily form hydroxide precipitates (e.g., Ca(OH)_2_ and Mg(OH)_2_) under alkaline conditions, which accumulate to cover the active sites of catalysts. Meanwhile, the high-concentration Cl^−^ ions trigger a competitive chlorine evolution reaction (ClER) at the anode, causing corrosion-induced degradation of electrode materials [145]. Constructing channeled porous Mo_2_C/B(CIP) catalysts to reduce precipitate clogging [146], or coating with Ni(OH)_2_ membranes that block Ca^2+^ and Mg^2+^ while allowing H_2_O and OH^−^ to pass through [147], are both strategies that enable the development of catalysts suitable for seawater conditions, gradually making the practical application of HERs feasible.

## Figures and Tables

**Table 1:** Comparison of the electrocatalytic activities of non-noble mental catalysts for HERs.

Catalysts	Electrolyte	Current Density [mA cm^−2^]	Overpotential [mV]	Tafel Slope [mV dec^−1^]	Stability [h]	Refs.
Ni_3_N-CeO_2_/NF	1 M KOH	10	30	42.79	35	[36]
NiO-Ni_3_Se_4_/MXene	1 M KOH	10	50	42.9	50	[42]
Co_2_P-MoNiP/NF	1 M KOH	10	46	49.3	100	[49]
NiO_x_ NTAs/CoP NSs	1 M KOH	10	51	56	50	[21]
Mn_1_-Ni_1_-Co_1_-P/NF	1 M KOH	10	14	58	50	[54]
A-MoP@PC	0.5 M H_2_SO_4_	10	68	41	40	[57]
CoP/DCS	1 M KOH	10	88	56	24	[58]
C@NiCoP/NF	1 M KOH	10	91	75.25	36	[59]
MoS_2_ nano island	0.5 M H_2_SO_4_	40	320	84	10	[60]
Co-MoS_2_@CN	0.5 M H_2_SO_4_	10	137	46.5	24	[61]
S_2_Mo-MoS_2_	0.5 M H_2_SO_4_	10	169	56	—	[62]
defect-rich 1T-2H MoS_2_/CFP	0.5 M H_2_SO_4_	10	192	44	38	[66]
SV-2H-MoS_2_	0.5 M H_2_SO_4_	10	369	78.4	—	[67]
Co-1T-MoS_2_-bpe	1 M KOH	10	118	83	50	[71]
V-MoS_2_	1 M KOH/0.5 m H_2_SO_4_	10	35/54	34/59	—	[74]
A-MoS_2_-Ni_3_S_2_-NF	1 M KOH/0.5 m H_2_SO_4_	10	145/95	79.9/107	12	[76]
P-CoMo_2_S_4_/Co_4_S_3_-Co_2_P	1 M KOH	10	54	61	40	[83]
Ni_3_S_2_/NiO	1 M KOH	10	95	84	25	[84]
Ni_3_S_2_/NiO nanomeshes	1 M KOH	10	73	127.2	50	[78]
NiSe/Ni_3_Se_2_-Fe-5	1 M KOH	10	144	77	72	[87]
CoMnTe_2_	1 M KOH	10	120	69	25	[88]
Ni-WO_2_/NF	1 M KOH	10	41	47	100	[91]
Ni_4_W/WO_x_	1 M KOH	10	22	32	60	[93]
Co@NiMoO-Ni/NF	1 M KOH	10	18	27	24	[3]
P-CoNiO_2_@NC	1 M KOH/0.5 m H_2_SO_4_	10	64.6/96.4	55/66	100	[94]
BaMoO_3_	1 M KOH	10	336	110	—	[96]
CoS_2_/WS_2_	0.5 M H_2_SO_4_	10	79	52	50	[103]
CoNi@CNC-500	1 M KOH	10	83	80	100	[105]
Fe_2_P-Co_2_P/NPC	1 M KOH	10	38	46.8	1000	[108]
Co_7_Fe_3_/Co	1 M KOH	10	68	55.8	100	[113]
Co-Ni@CC	0.5 M H_2_SO_4_	10	231	111.7	—	[114]
NiW	1 M KOH	500	198	117	200	[115]
NiFeAl/NF	1 M KOH	10	31	37	100	[117]
FeCoNiMnZn/N-CNTs-FH	1 M KOH	10	184	112	50	[121]
HEOs/NiFeCuMoMn	1 M KOH	10	50.5	70.46	50	[124]
MoO_2_/Ni_3_S_2_/NF	1 M KOH	10	70.4	85.2	24	[126]
Cu,Co/NSC1	1 M KOH	10	159	75.9	25	[134]
Co@CN_x_	0.5 M H_2_SO_4_	10	270	126	—	[135]
Ni@NCW	0.5 M H_2_SO_4_	10	158	75	18	[138]

## Data Availability

No new data were created or analyzed in this study.
